# Combined examination of sequence and copy number variations in human deafness genes improves diagnosis for cases of genetic deafness

**DOI:** 10.1186/1472-6815-14-9

**Published:** 2014-09-10

**Authors:** Haiting Ji, Jingqiao Lu, Jianjun Wang, Huawei Li, Xi Lin

**Affiliations:** 1 Department of Otolaryngology, Eye & ENT Hospital, Fudan University, #83 Fenyang Road, Shanghai 200031, P.R China; 2 Department of Otolaryngology, Emory University School of Medicine, 615 Michael Street, Atlanta, GA 30322-3030, USA

**Keywords:** Genetic deafness, Copy number variations, Sequence mutations, Next-generation sequencing, Deafness gene panel, Hearing

## Abstract

**Background:**

Copy number variations (CNVs) are the major type of structural variation in the human genome, and are more common than DNA sequence variations in populations. CNVs are important factors for human genetic and phenotypic diversity. Many CNVs have been associated with either resistance to diseases or identified as the cause of diseases. Currently little is known about the role of CNVs in causing deafness. CNVs are currently not analyzed by conventional genetic analysis methods to study deafness. Here we detected both DNA sequence variations and CNVs affecting 80 genes known to be required for normal hearing.

**Methods:**

Coding regions of the deafness genes were captured by a hybridization-based method and processed through the standard next-generation sequencing (NGS) protocol using the Illumina platform. Samples hybridized together in the same reaction were analyzed to obtain CNVs. A read depth based method was used to measure CNVs at the resolution of a single exon. Results were validated by the quantitative PCR (qPCR) based method.

**Results:**

Among 79 sporadic cases clinically diagnosed with sensorineural hearing loss, we identified previously-reported disease-causing sequence mutations in 16 cases. In addition, we identified a total of 97 CNVs (72 CNV gains and 25 CNV losses) in 27 deafness genes. The CNVs included homozygous deletions which may directly give rise to deleterious effects on protein functions known to be essential for hearing, as well as heterozygous deletions and CNV gains compounded with sequence mutations in deafness genes that could potentially harm gene functions.

**Conclusions:**

We studied how CNVs in known deafness genes may result in deafness. Data provided here served as a basis to explain how CNVs disrupt normal functions of deafness genes. These results may significantly expand our understanding about how various types of genetic mutations cause deafness in humans.

## Background

Hearing impairment is one of the most common human disabilities. Multiple factors, including ototoxic drug usage, noise exposure, and genetic mutations can cause hearing loss. The majority of human hearing loss cases (~70%), however, are attributed to genetic factors [1]. Large numbers of studies have identified many deafness genes whose functions are essential for normal hearing [2] (Additional file 1: Table S1). Deleterious DNA sequence mutations in these genes lead to inherited deafness. In most cases a single monogenic mutation is sufficient to result in severe hearing loss [2,3]. The deafness genotype and phenotype relations are usually tightly defined [1], making information about genetic mutations valuable in providing essential diagnostic and prognostic information. The diagnostic information could be used in the management and treatment of hearing loss as it identifies the root cause of deafness.

Mutations that cause congenital deafness involve a highly diverse group of genes [4]. Traditionally, genetic diagnosis of deafness genes is done by Sanger sequencing one gene at a time. In recent years, methods based on the NGS approach have been developed to sequence multiple deafness genes in one test, which have greatly increased the efficiency in screening for large numbers of mutations in deafness genes for diagnostic purposes as well as for finding new deafness genes [3,5]. However, NGS screening of sequence mutations in multiple genes are usually limited to detecting single nucleotide variations and small insertions and deletions (InDels) usually smaller than 20 base pairs (bps). Copy number variations (CNVs) are copy number gains or losses of DNA segments ranging from 100 to more than a million base pairs in length. CNVs are important factors for human genetic and phenotypic diversity [6]. CNVs are currently not analyzed by conventional data analysis methods in the NGS approach to study deafness. It is estimated that up to 29% of the genomes of unrelated individuals may differ in copy numbers [6]. Many CNVs have been associated with either resistance to diseases or identified as the cause of diseases [6]. However, contribution of CNVs in genetic deafness is unclear. Here we have used a NGS-based approach to investigate novel mechanisms of combined sequence variations and CNVs in 80 deafness genes that cause inherited deafness.

## Methods

### Patient history and physical/laboratory examinations

Patients (N = 79) were recruited from outpatients who visited the Eye & ENT Hospital of Fudan University. All participants were informed about the scope and requirements of the study, and signed the patient consent form approved by the ethics committee of Fudan University. They were all diagnosed with sensorineural hearing loss. Cases caused by trauma, use of ototoxic drugs, and otitis media were excluded from this study. Patients with history of meningitis and maternal Cytomegalovirus (CMV), if they are known, were also excluded. Patient history information included the time of onset of hearing loss, and the degree of hearing loss, classified as either mild (21–40 dB, N = 14), moderate (41–70 dB, N = 35), severe (71–90 dB, N = 11), or profound (>90 dB, N = 19). Other pertinent facts included family history of deafness, pregnancy and labor history, general health conditions, and chronic diseases that might affect hearing such as type 2 diabetes mellitus and hypertension. General physical examinations were conducted with particular emphasis on examination of the ears, intellectual ability, syndromic features of congenital deafness (such as the presence of white hair at the forehead, hyperpigmentation, heterochromia iridis, dystopia canthorum, and broadening of the nasal root). Laboratory tests included pure tone audiometry, acoustic immittance, auditory brainstem responses, multiple-frequency auditory steady-state responses, and distortion product otoacoustic emission (DPOAE). Computed tomography and magnetic resonance imaging were conducted for some patients for whom it was considered clinically necessary.

### Protocols for DNA extraction, PCR-based Sanger sequencing for detecting mutations in validation tests

Genomic DNA (gDNA) was isolated from 3–5 ml of blood, according to the standard protocol provided by the manufacturer (Qiagen, Valencia, CA). The quality of gDNA was assured by examining the optical density ratio (260/280 ratio) and by checking the results via gel electrophoresis imaging. The coding exon of *GJB2* (exon2) was amplified by polymerase chain reactions (PCR) using two specific primers: F (Fn1): 5’ TTGGTGTTTGCTCAGGAAGA 3’, R (R3C): 5’ GGCCTACAGGGGTTTCAAAT 3. The reaction was performed in a volume of 25 μl containing 1.5 mM MgCl_2_ 250 mM dNTPs, 1 mM of each primer, 100 ng of genomic DNA, and 1.25 U of rTaqDNA polymerase with an initial denaturing step at 95.8°C for 12 min, followed by 35 cycles at 94.8°C for 45 s, 60.8°C for 1 min, 72.8°C for 1.20 min, and a final elongation at 72.8°C for 7 min. Predicted amplicon sizes were confirmed by agarose gel electrophoresis. Mutations in *GJB2* were examined by the standard Sanger sequencing method by a commercial source (Beckman Coulter Genomics, Danvers, MA).

### NGS protocol and data analysis

High-molecular weight gDNA (5 μg) was fragmented ultrasonically with the Covaris E210 DNA shearing instrument (Covaris Inc., Woburn, MA) to an average size of 300 bps for subsequent construction of Illumina NGS libraries. The Covaris protocol is set at 3 minutes total duration, duty cycle 10%, intensity 5, and 200 cycles per burst. Fragmented gDNA libraries for sequencing on the Illumina platform (HiSeq2000) were prepared with the NEBNext™ DNA Sample Prep Master Mix set (E6040, NEB Biolab, Ipswich, MA). End repair of DNA fragments, addition of a 3’ adenine (A), adaptor ligation, and reaction clean-up were carried out following the manufacturer’s protocol. gDNA libraries were cleaned and size selected using the AMPure DNA Purification kit (Beckman Agencourt, Danvers, MA). The ligated product (20 ng) was amplified for 14 PCR cycles with Illumina PCR primers InPE1.0 and indexing primer following the manufacturer’s instructions. The PCR products were purified again with QIAquick MinElute column and eluted into 50 μl of hybridization buffer (HB, Roche NimbleGen, Madison, WI).

Targeted capture of deafness genes (Additional file 1: Table S1) and the examination for the enrichment of these genes by quantitative PCR (qPCR) were carried out by protocols described in our published paper [7]. Systematic comparison of different tools in population-scale genomic CNV analysis found notable differences between different methods used in identifying genomic regions ascertained, size-range and breakpoint [8]. Since only partially-overlapping CNVs are identified using different methods, some studies used as many as 36 CNV call-sets to help improve the accuracy of finding high-confidence CNVs [8]. In our study, FASTQ data files generated after sequencing with the Illumina HiSeq2000 were processed using four independent bioinformatic data processing pipelines, since the use of multiple independent bioinformatic pipelines help improve accuracy in finding high-confidence CNVs [9]. These bioinformatic platforms are:

(1) A web-based open source platform called Galaxy (https://usegalaxy.org/), which runs on a local Linux server. The tools we used on Galaxy Platform were: (a) BWA (ver. 0.7.4), to generate SAM (Sequence Align/Map) files; (b) Samtools (ver.0.1.19), which were used to transform Binary SAM into BAM and were sorted with samtools; (c) Picard (ver. 1.79), this was used to remove PCR duplicates in the sorted BAM files; (d) GenomeAnalysisTK-1.6 (GATK-1.6). The duplicate-removed BAM files were used as inputs of GATK-1.6 for InDel re-alignment and base quality recalibration using known InDels from dbSNP137 and the 1000 Genome project. Target region coverage and VCF (Variant Calling Format) files of SNP/InDel calling were generated by GATK, based on processed BAM files.

(2) A software package developed at the Broad Institute called Genome Analysis Toolkit (GATK) (http://www.broadinstitute.org/gatk/).

(3) A paid commercial bioinformatic analysis platform (http://www.dnanexus.com).

(4) A proprietary bioinformatic processing pipeline called NGSeq, developed in-house specifically for analyzing mutations in targeted deafness genes.

The variation and CNV results presented here are consensus results of the four bioinformatic pipelines. The consensus rates among different platforms were high, with the lowest being 94.5% between the GATK and Galaxy pipelines. Sequencing results obtained by the NGS method for the coding region of the *GJB2* gene were compared to those obtained by the Sanger method (N = 8) for validation purposes. A high percentage (99.2%) of detected sequence variations (SNPs) in the *GJB2* gene as identified by the two methods agreed with each other. All samples processed in this project had an on-target average coverage of greater than 100 (Additional file 1: Table S1). Distribution of average coverage (n = 10) at 0x, 1x, 10x & 20x was 1.1 ± 0.5, 4.8 ± 1.1, 5.6 ± 1.9 and 11.2 ± 2.9, respectively. After controlling for data quality (coverage ≥20 and Phred-like quality score ≥30), we obtained the VCF reports for the coding regions and the exon-intron boundaries of the targeted deafness genes (Additional file 1: Table S1). Variations contained in VCF reports were filtered by a custom knowledge database in order to identify candidate disease-causing mutations. Information in this database was collected from the following sources: (1) Human Genome Mutation Database (HGMD); (2) data from unpublished mutation and normal hearing control results obtained in the authors’ lab; (3) data made from consensus predications of both PolyPhen and Shift bioinformatic algorithms [10]. Variants with high population allele frequencies (>0.02), as reported by data collected from the 1000 genome project, were filtered out. The identified variations were classified into nine categories (see Additional file 2: Table S2). Mutations in these categories can be defined as disease-causing, highly likely to be disease-causing, predicted but unconfirmed to be disease-causing, carrier of mutations, and novel mutations with low population allele frequencies (less than 0.005) of unknown significance.

In the hybridization step for capturing the targeted deafness genes, we normally hybridized 20–30 samples together. Samples were differentiated by different barcodes. In-house generated cDNA capture probes with lengths ranging from 100 to ~5000 bps were used. Biotinylated probes were hybridized overnight with fragmented genomic DNA (gDNA) at 47°C in a thermocycler (Bio-Rad T100, Hercules, CA). Captured gDNA fragments were enriched using streptavidin dynabeads (Beckman Coulter, Brea, CA). For targeted NGS projects, on-target coverage after the capturing step is a more appropriate index for describing the quality of sequencing, not the number of overall reads. The average depth of coverage for each exon of targeted deafness genes was used in the calculation of CNVs. Compared to previous methods used to detect CNVs from whole human genome or exome NGS data [9], this is a novel method used to specifically detect CNVs from NGS data obtained in a disease (e.g., deafness) panel. The mean coverage can be affected by CNVs, the relative quantity of DNA in each sample loaded for hybridization, and the experimental conditions. By analyzing the CNVs only for samples that were hybridized together, we could control the level of variation introduced by the experimental conditions. The relative quantity of loading DNA for each sample was normalized by the summed total of the average depth of coverage (S_d_), which yielded a normalized average depth of coverage N_adc_ = (depth of coverage of specific exon)/S_d_. The ratio of CNVs (R_CNV_) for each exon of the deafness genes was calculated by the following equation:

$$\begin{array}{l} R_{\mathrm{CNV}}=N_{\mathrm{adc}}/(\mathrm{average}\;N_{\mathrm{adc}}\mathrm{of}\;\mathrm{all}\;\mathrm{other}\;\mathrm{samples}\;\mathrm{in}\;\mathrm{the}\\ \;\mathrm{same}\;\mathrm{hybridization}\;\mathrm{for}\;\mathrm{this}\;\mathrm{exon}) \end{array}$$

**Figure 1:**
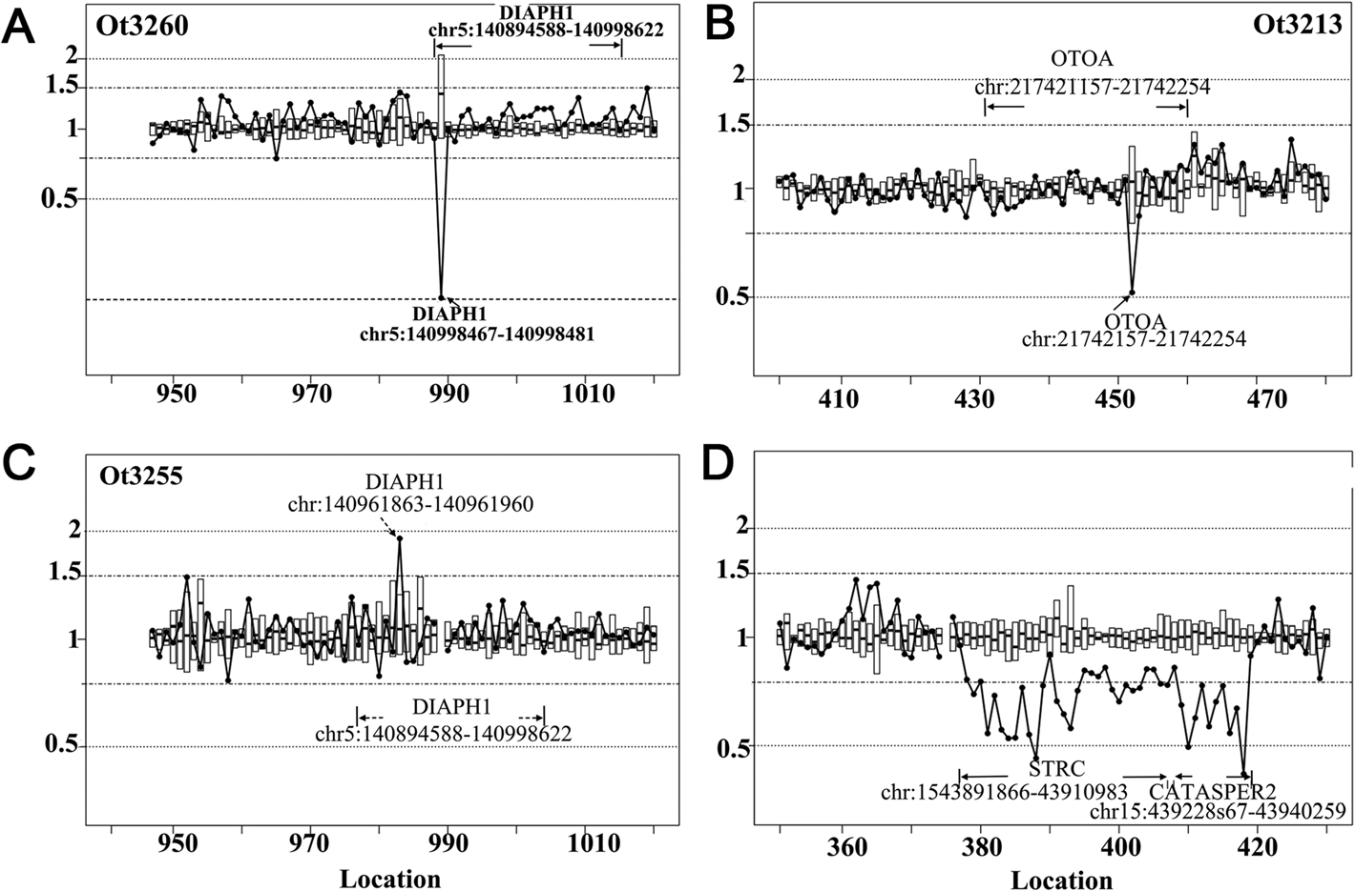
**Typical examples of CNVs detected in patient samples.** The x-axis is the exon location sequentially arranged for the targeted deafness genes as listed in the Additional file 1: Table S1. The y-axis gives the R_CNV_ ratio calculated for CNVs. **(A)** An example of homozygous deletion of the exon 1 of the *DIAPH1* gene. **(B)** This example shows a heterozygous deletion of the exon 20 of the *OTOA* gene. **(C)** An example of a CNV gain of exon 8 of *DIAPH1* gene. The ratio of exon 8 of *DIAPH1* gene was close to 2, suggesting that the DNA segment containing this exon was doubled compared to the normal copy number. **(D)** This example shows heterozygous deletion covering two adjacent genes, *STRC* and *CATSPER2* on Chromosome 15 and span at least 48 k bps.

For normal copy numbers (two copies) of any exons, the ratio should be close to one. Heterozygous and complete deletions would result in a ratio close to 0.5 and 0, respectively. CNV gains would yield ratios of 1.5 or larger (illustrated in Figure 1). The resolution of our method used to detect CNVs is the size of a single exon as determined by comparing the average depth of coverage for each exon. Bioinformatic analysis of the gene structure showed that the average exon size for the 80 deafness genes (Additional file 1: Table S1) is 4491 bps.

In order to validate our NGS-based approach in identifying the CNVs, we randomly selected 15 putative CNVs and examined these CNVs again using the independent qPCR approach. A commercially-available CNV test kit based on SYBR green chemistry (DyNAmo ColorFlash SYBR Green qPCR Kit, Thermo Scientific Inc., Pittsburgh PA) was used. A house-keeping gene (*GAPDH*) was used as a reference for the qPCR quantification. Primers for amplifying selected genes and reference genes (Additional file 3: Table S4) were designed by Primer3 (http://frodo.wi.mit.edu). Triplicate qPCR reactions were conducted for each sample, each using a reaction volume of 25 μl. The relative abundance of genomic DNA in samples was calculated by looking at the differences in the cycle numbers obtained in the linear increasing phase, using the following steps:

1) Calculating threshold cycle (Ct) differences between the target gene and the reference gene

$$\mathrm{\Delta C}t_{\left(\mathrm{calibrator}\right)}=Ct_{\left(\mathrm{target},\;\mathrm{calibrator}\right)}–Ct_{\left(\mathrm{reference},\;\mathrm{calibrator}\right)}$$

$$\mathrm{\Delta C}t_{\left(\mathrm{test}\right)}=Ct_{\left(\mathrm{target},\;\mathrm{test}\right)}–Ct_{\left(\mathrm{reference},\;\mathrm{test}\right)}$$

2) Normalizing ΔCt of the test samples to the ΔCt of the calibrator:

$$\mathrm{\Delta \Delta Ct}=\mathrm{\Delta Ct}\left(\mathrm{test}\right)–\mathrm{\Delta Ct}\left(\mathrm{calibrator}\right)$$

3) Calculating the CNV ratio using following formula: *CNV ratio* = 2^− *ΔΔCt*^

## Results

### Analysis of DNA sequence variations and deafness mutations by the NGS approach

In the 79 sporadic cases of patients clinically diagnosed with sensorineural hearing loss, we were able to identify 16 patients with previously-reported pathogenic mutations (Category I as defined in the Additional file 2: Table S2) and 5 more with novel likely-pathogenic mutations (Category III). Details on the DNA sequence mutations we found are given in Table 1. In addition, we found 8 possible disease-causing mutations using the predications independently agreed upon by both SIFT and PolyPhen bioinformatic algorithms [10,11]. The two algorithms both predicted these mutations as damaging or probably damaging to protein functions of deafness genes known to be essential for hearing (Additional file 1: Table S1).

**Table 1:** Previously-reported and likely pathogenic deafness mutations found in 79 patient samples examined in this study

**Category**	**# of occurrence**	**SampleID**	**Gene name**	**Position on Chr**	**Mutation**
Category I	16	Ot3271	GJB2	Chr13: 20763612 C > T	NM_004004:exon2: c. G109A: p. V37I
		Ot3275	WFS1	Chr4: 6303680 A > G	NM_001145853:exon8: c. A2158G: p. I720V, NM_006005:exon8: c. A2158G: p. I720V
		Ot3212	GJB2	Chr13: 20763486 del G	NM_004004:exon2: c.235delC: p. L79fs
		Ot3213	SLC26A4	Chr7: 107323898 A > G	NM_000441:exon8: c.919-2A > G
		Ot3227	GJB2	Chr13: 20763612 C > T	NM_004004:exon2: c. G109A: p. V37I
		Ot3230	SLC26A4	Chr7: 107323898 A > G	NM_000441:exon8: c.919-2A > G
		Ot3241	GJB2	Chr13: 20763612 C > T	NM_004004:exon2: c. G109A: p. V37I
		Ot3242	GJB2	Chr13: 20763612 C > T	NM_004004:exon2: c. G109A: p. V37I
		Ot3252	GJB2	Chr13: 20763612 C > T	NM_004004:exon2: c. G109A: p. V37I
		Ot3255	GJB2	Chr13: 20763486 del G	NM_004004:exon2: c.235delC: p. L79fs
		Ot3256	SLC26A4	Chr7: 107323898 A > G	NM_000441:exon8: c.919-2A > G
		Ot3258	SLC26A4	Chr7: 107323898 A > G	NM_000441:exon8: c.919-2A > G
		Ot3260	GJB2	Chr13: 20763486 del G	NM_004004:exon2: c.235delC: p. L79fs
		Ot3266	SLC26A4	Chr7: 107323898 A > G	NM_000441:exon8: c.919-2A > G
		Ot3276	GJB2	Chr13: 20763486 del G	NM_004004:exon2: c.235delC: p. L79fs
		Ot3284	SLC26A4	Chr7: 107323898 A > G	NM_000441:exon8: c.919-2A > G
Category III	5	Ot3226	DSPP	Chr4: 88537078 ins CGATAGCAG	NM_014208:exon5: c.3264_3265insCGATAGCAG:p. S1088delinsSRX
		Ot3243	DSPP	Chr4: 88537270 ins TAGCAGCGATAGCAGCGA	NM_014208:exon5: c.3456_3457insTAGCAGCGATAGCAGCGA:p. D1152delinsDX
		Ot3256	DSPP	Chr4: 88537078 ins CGATAGCAG	NM_014208:exon5: c.3264_3265insCGATAGCAG:p. S1088delinsSRX
		Ot3266	DSPP	Chr4: 88537078 ins CGATAGCGG	NM_014208:exon5: c.3264_3265insCGATAGCGG:p. S1088delinsSRX
		Ot3270	DSPP	Chr4: 88537078 ins CGATAGCAA	NM_014208:exon5: c.3264_3265insCGATAGCAA:p. S1088delinsSRX
Category V	1	Ot3209	ESRRB	Chr14: 76905712 A > G	NM_004452:exon4: c. A16G: p. R6G
Category VII	7	Ot3217	DSPP	Chr4: 88537088 A > G	NM_014208:exon5: c. A3274G: p. N1092D
		Ot3242	COCH	Chr14: 31358873 A > G	NM_001135058:exon11: c. A1529G: p. K510R, NM_004086:exon12: c. A1529G: p. K510R
		Ot3260	DSPP	Chr4: 88537088 A > G	NM_014208:exon5: c. A3274G: p. N1092D
		Ot3268	MYO6	Chr6: 76576249 A > G	NM_004999:exon17: c. A1681G: p. R561G
		Ot3270	DSPP	Chr4: 88537088 A > G	NM_014208:exon5: c. A3274G: p. N1092D
		Ot3284	SOX2	Chr3: 181430197 A > G	NM_003106:exon1: c. A49G: p. T17A
		Ot3284	DSPP	Chr4: 88537088 A > G	NM_014208:exon5: c. A3274G: p. N1092D

The definition of each category is given in the Additional file 2: Table S2.

We identified many well-established deafness mutations (e.g., *GJB2* c.235delC, *GJB2* p.V37I, *SLC26A4* c.919-2A > G [12], as well as previously un-reported novel mutations (e.g., *DSPP* c.3264_3265insCGATAGCGG, p.S1088delinsSRX) which we predict to be deleterious to protein functioning. The predictions were based on the presence of frameshift or premature stop codon mutations, which disrupt the function of genes known to be essential for hearing [13]. In summary, depending on the criteria used, the genetic basis of 27-37% of the sporadic cases of sensorineural hearing loss was likely attributed to DNA sequence mutations (Table 1). The nine most common deafness genes found in these patients were (in the order of occurrence, from high to low): *GJB2, DSPP, SLC26A4, COCH, ESRRB, MYO6, SOX2, TMPRSS3* and *WFS1*.

### CNVs found by the NGS approach and validation results by qPCR

Among the 80 deafness genes (total of 1253 exons) analyzed from the 79 patient samples, we identified a total of 97 CNVs. These included 72 CNV gains and 25 CNV losses (details are given in the Table 2 and Additional file 4: Table S5). Figure 1 gives examples of the CNV losses (Figure 1, A, B&D) and gains (Figure 1C) we detected. The x-axis is the exon location sequentially arranged for the deafness genes listed in the Additional file 1: Table S1. The sequential number, corresponding exon number, and gene name can be found in Additional file 1: Table S1. The y-axis in Figure 1 is the ratio of CNVs (R_CNV_), as calculated by the formula given in the Methods section. Samples with normal copy number (N = 2) at the targeted exon location would give a ratio near one. The average ratio and range of standard deviation for each exon of deafness gene are given by the box plot in the graph (Figure 1). Figure 1A shows an example of a homozygous deletion of the exon 1 of the *DIAPH1* gene, which could directly disrupt gene function. Figure 1 B & D shows examples of heterozygous deletions. While the deletion shown in Figure 1B affected only one exon (exon 20 of the *OTOA* gene), the example shown in Figure 1D is a much larger CNV deletion that affected two adjacent genes (*STRC* and *CATSPER2* on the Chromosome 15) and spans at least 48 k bps. The example in Figure 1C shows a CNV gain. The ratio of exon 8 of the *DIAPH1* gene was close to 2, suggesting that the DNA segment containing this exon was doubled compared to the normal copy number.

**Table 2:** Types of CNVs found in the 79 patient samples and their predicted consequences in causing deafness

**Types of CNV**	**Number of occurrence**	**Possible zygosity**	**Disease-causing?**
Homozygous deletion (copy# ratio = 0)	4	Homozygous (Hom)	Yes
Heterozygous deletion (copy# ratio = 0.5)	21	Heterozygous (Het)	Possible when combined with sequence mutation(s)
CNV increase with copy# ratio = 1.5	27	Heterozygous	Possible when combined with sequence mutation(s)
CNV increase with copy# ratio = 2	31	Het/Hom	Possible
CNV increase with copy# ratio > 2	14	Het/Hom	Possible

Among the 79 patients we studied, the total number of deafness genes affected by CNVs is twenty seven. Among these 27, only five have corresponding pseudogenes. We detected the most number of CNVs on the *DIAPH1* gene, which is located on Chromosome 5. Other affected deafness genes and the frequency of occurrence for each gene are given in the Table 3. Most (approximately 97.6%) of the CNVs we identified affected only one exon. CNV identifications made using the NGS approach were verified independently using the qPCR method. Among 15 randomly selected CNVs, we found that qPCR confirmed most (14 out of 15) of the CNVs found by the NGS method (Additional file 5: Table S3), therefore suggesting that the ratios derived from the average read depth of the NGS data provided a reliable estimate of the CNVs in deafness genes.

**Table 3:** The CNVs and the rank of occurrences found in targeted deafness genes among the 79 patient samples

**Gene name**	**Chromosome number**	**# of occurrence of CNVs**
*DIAPH1*	Chr5	16
*COL11A2*	Chr6	15
*MYO1C*	Chr17	12
*STRC*	Chr15	8
*OTOA*	Chr16	6
*MYO3A*	Chr10	5
*ERCC2*	Chr19	4
*USH1C*	Chr11	4
*GRHL2*	Chr8	3
*MYO6*	Chr6	3
*CDH23*	Chr10	2
*MYH14*	Chr19	2
*MYO15A*	Chr17	2
*MYO7A*	Chr11	2
*SOX2*	Chr3	2
*COCH*	Chr14	1
*COL9A3*	Chr20	1
*CATSPER2*	Chr15	1
*DFNA5*	Chr7	1
*DFNB59*	Chr2	1
*EYA4*	Chr6	1
*LHX3*	Chr9	1
*MTAP*	Chr9	1
*OTOR*	Chr20	1
*SLC26A4*	Chr7	1
*SLC26A11*	Chr17	1
TMPRSS5	Chr11	1
GJB3	Chr1	1

### Combined sequence variation and CNV analysis for the cause of genetic deafness

Results presented in Table 1 show that, under the most relaxed criteria, we could only detect pathogenic genetic mutations in 29 of the 79 samples. We found another 20 patients who only showed CNVs without any point mutations. Four of the patients (patient_ID: J246, J256, J267, J319) carried homozygous CNV deletions that may directly disrupt the functions of deafness genes. We also found 12 patients to be carriers of deafness mutations (belonging to the categories II or IV as defined in Additional file 2: Table S2), who also bear CNVs (Additional file 6: Table S6). One patient (ot3285) had a heterozygous deafness mutation and a copy number reduction in the same gene (*GJB3,* Additional file 4: Table S5). Since *GJB3* has only one coding exon, this combination may potentially result in compounded effects which cause disruption of *GJB3* protein functions, leading to deafness. The other 11 carriers had CNVs affecting deafness genes different from those affected by DNA sequence mutations, and the significance of such combinations is currently unknown. Overall, combined sequence variation and CNV analysis increased the detection rate from 36.7% (29/79) to 43.0% (34/79) among 79 sporadic patients clinically diagnosed with sensorineural hearing loss.

## Discussion

Genetic tests performed in the past, whether referring to the traditional Sanger method or the newer NGS method, only detect DNA sequence point substitutions and small InDel mutations (usually less 20 bps) [4]. These types of mutations have been well established as the major cause of congenital deafness. With the availability of genome-wide sequencing data and other genotyping technologies (e.g., arrays for comparative genomic hybridization (aCGH arrays)), large numbers of structural variations, including CNVs (e.g., >38,000 CNVs found in segments >100 bp in size), balanced inversions, and translocations have been reported [6]. In terms of total bps involved, the incidence of structural variations is estimated to be much higher than that of SNPs in populations [14]. The contribution of CNVs to genetic deafness only begins to be revealed recently in a few papers that have addressed this specific topic [15,16]. We have evaluated the impact of CNVs in 80 targeted deafness genes (Additional file 1: Table S1) on inherited hearing loss. We performed a combined analysis of DNA sequence mutations and CNVs to assess how protein functions known to be essential for hearing may be disrupted. We were able to identify 21 out of 79 patients bearing disease-causing sequence mutations (Table 1). For the remaining samples where we could not find sequence mutations directly responsible for deafness, we identified an additional four patients bearing homozygous CNV deletions which may directly disrupt gene function. Additionally, we found another patient bearing both sequence and CNV mutations in the same gene, which could unmask the heterozygous effects of a *GJB3* point mutation and harm gene function. The high incidence of DSPP variants we found in category III (Table 1) is unlikely to be caused by platform-specific errors, because we didn’t find similar results in other batches of samples we processed (unpublished data). Those samples included more than 1000 patients diagnosed with sensorineural hearing loss and about 800 normal-hearing control samples.

Recent advances in technology have provided powerful tools for the detection and analysis of CNVs at either the whole-genome level or for targeted loci. Commonly used methods in previous studies are either array-based or polymerase chain reaction (PCR) based methods. So far, aCGH arrays have been the most widely used method for identification of CNVs on the genomic scale. In the aCGH method, the ratios of respective signal intensities derived from the test to those of reference samples are compared to give a measure of the CNVs. The major disadvantage of aCGH arrays is its poor resolution of CNVs, which is generally much larger than the size of a single exon as achieved in this work. Quantitative PCR (qPCR) is another commonly used method for screening targeted genomic regions for CNVs, which is especially efficient in detecting CNVs at single loci. The qPCR approach has the advantage of potentially avoiding interference of pseudogenes if amplification primers are carefully designed. However, major disadvantages of qPCR detections are that (1) qPCR is unable to give precise integer measurements of CNVs [17]; (2) it is unable to efficiently process multiple loci/genes for CNV analysis in one test. Recently, a new approach has been developed by taking advantage of the depth of read coverage generated in the NGS data analysis of each base pair in the targeted regions [9,18], which is similar to what was used in this study. Most published methods on CNV detections from NGS data are designed for examining CNVs on the whole genome or whole exome scale, with specific mathematical models applied in the algorithms [9]. For example, JointSLM and ExoCNVTest are designed to detect common CNVs shared among many samples. CoNIFER and XHMM are made to detect rare CNVs using population data. In our approach, we used the average read-depth of samples hybridized together for exons covered, which greatly reduced the variations introduced by GC content, capture efficiency, and alignment biases (Figure 1). The optimal detection length for CNVs also depends on the specific program used. Pindel is effective for the detection of small deletions less than 300 bp. In contrast, most prior read-depth based bioinformatic tools are used to identify large CNVs (e.g., >10,000 bps). The resolution of our method of detecting CNVs is the size of single exon, which varies between about 100 to a few thousand bps. The average size of targeted exons for the 80 deafness genes is 4491 bps (Additional file 1: Table S1). Drawbacks of our approach include the inability to detect the breakpoints in CNVs, as well as common CNVs presented in many samples. We used the average coverage of targeted exons of deafness genes as the basis to calculate CNVs in the targeted regions. Another vulnerability is that the presence of pseudogenes may cause false CNV gain events. Among the 27 genes that showed CNVs (Table 3), we found that five of them have corresponding pseudogenes (*MTAP, MYO15A, OTOAn SOX2* and *STRC*), according to database http://www.pseudogene.org. The CNV data of these small group of genes (5 out of 80), especially those on CNV gains including qPCR validation results, need to be cautiously interpreted.

We are only at an early stage of understanding the combined effects of CNVs and mutations in the deafness genes, especially about polymorphisms in CNVs in the normal hearing control subjects. Similar to single nucleotide polymorphisms (SNPs), most CNVs represent benign copy number polymorphisms, and past studies show that many of them have a population frequency of >1% [14,19]. Deleterious CNVs include homozygous deletions of coding exons, which disrupt protein functions, and therefore are highly likely to cause deafness. The meaning of CNV gains, however, is more complicated to interpret. Nonetheless, this work has demonstrated that genetic causes of deafness could be found in a higher percentage of patients when considering a combined analysis of both DNA sequence variations and CNVs.

## Conclusions

We investigated how CNVs in 80 known deafness genes may result in deafness. Data presented in this paper suggest that CNVs are a major type of structural variation, which affect the function of genes known to be essential for normal hearing, and thus play a significant role in causing deafness. Among 79 sporadic cases clinically diagnosed with sensorineural hearing loss, we identified both disease-causing DNA sequence mutations that were previously reported, and novel CNV gains and losses in 27 deafness genes. These CNVs included homozygous deletions, which may directly give rise to deleterious effects on protein functions known to be essential for hearing, as well as heterozygous deletions and CNV gains, which when compounded with sequence mutations in deafness genes could potentially harm gene functions known to be essential for hearing. The data provided here serve as a basis to explain how CNVs could disrupt normal functions of deafness genes. With further accumulation of more diverse types of samples, the analysis in CNVs may significantly expand our understanding about how combined DNA sequence and structural mutations cause deafness in humans, and ultimately could help to better explain the genetic causes of sensorineural hearing loss in patients.

## Abbreviations

CNVs: Copy number variations; qPCR: Quantitative polymerase chain reaction; UNHS: Universal newborn hearing screening; NGS: Next-generation sequencing; InDels: Insertions and deletions; gDNA: Genomic DNA; bps: Base pairs; PCR: Polymerase chain reaction; VCF: Variant call format; S_d_: Average depth of coverage; N_adc_: Normalized average depth of coverage; R_CNV_: Ratio of CNVs; aCGH: Arrays for comparative genomic hybridization; GATK: Genome analysis toolkit.

## Competing interest

The authors declare no conflicting financial and non-financial (e.g., political, personal, religious, academic, commercial or any other) interests.

## Authors’ contributions

HTJ conducted molecular genetic experiments, qPCR studies, analyzed and summarized the data, and wrote the paper. JQL performed sequence alignment, analyzed and summarized the data, and wrote part of the paper. JJW conducted experiments and analyzed molecular biology data. HWL participated in study coordination, analyzed data, and wrote part of the paper. XL conceived the study idea, participated in its design and coordination, conducted experiments, analyzed and summarized the data, and wrote the paper. All authors read and approved the final manuscript.

## Pre-publication history

The pre-publication history for this paper can be accessed here:

http://www.biomedcentral.com/1472-6815/14/9/prepub

## Supplementary Material

Additional file 1: Table S1 Gene names, genomic coordinates of exons and sequential numbers of deafness genes. Click here for file

Additional file 2: Table S2 Definition of nine categories of mutations/variants in targeted deafness genes detected by the NGS method. Click here for file

Additional file 3: Table S4 Primers used in real-time qPCR validation detection of CNVs. Click here for file

Additional file 4: Table S5 Clinical findings and summary of NGS results of 79 patients. Click here for file

Additional file 5: Table S3 Comparison of CNV results obtained by NGS and qPCR methods. Click here for file

Additional file 6: Table S6 Information of 12 patients who showed both point mutations & CNVs. Click here for file
