# Supplementary material for: Combined examination of sequence and copy number variations in human deafness genes improves diagnosis for cases of genetic deafness
Source: BMC Ear Nose Throat Disord. 2014 Sep 10;14:9. doi: 10.1186/1472-6815-14-9 (PMC4194081; doi:10.1186/1472-6815-14-9)
Supplement: Additional file 1: Table S1 — Gene names, genomic coordinates of exons and sequential numbers of deafness genes. [file 1472-6815-14-9-S1.pdf]

Supplemental Table 1. Gene names, genomic coordinates of exons and sequential numbers of deafness genes studied in this project.

Sequential#	location	Gene name
1	chr1:6485018-6485309	ESPN
2	chr1:6488288-6488479	ESPN
3	chr1:6500316-6500500	ESPN
4	chr1:6500688-6500868	ESPN
5	chr1:6500996-6501125	ESPN
6	chr1:6504543-6504742	ESPN
7	chr1:6505726-6505995	ESPN
8	chr1:6508703-6509151	ESPN
9	chr1:6511665-6511808	ESPN
10	chr1:6511895-6512156	ESPN
11	chr1:6517236-6517333	ESPN
12	chr1:6517379-6517476	ESPN
13	chr1:6520061-6520206	ESPN
14	chr1:35226858-35227656	GJB4
15	chr1:35250366-35251176	GJB3
16	chr1:41249768-41250079	KCNQ4
17	chr1:41282934-41283032	KCNQ4
18	chr1:41283838-41283962	KCNQ4
19	chr1:41284179-41284352	KCNQ4
20	chr1:41285021-41285144	KCNQ4
21	chr1:41285549-41285657	KCNQ4
22	chr1:41285837-41285934	KCNQ4
23	chr1:41287982-41288080	KCNQ4
24	chr1:41289771-41289930	KCNQ4
25	chr1:41296758-41296976	KCNQ4
26	chr1:41298688-41298785	KCNQ4
27	chr1:41300641-41300770	KCNQ4
28	chr1:41303339-41303466	KCNQ4
29	chr1:41303985-41304195	KCNQ4
30	chr1:55464862-55465036	BSND
31	chr1:55470694-55470792	BSND
32	chr1:55472672-55472945	BSND
33	chr1:55473889-55474301	BSND
34	chr1:160011185-160012322	KCNJ10
35	chr2:26680910-26681088	OTOF
36	chr2:26682895-26683073	OTOF
37	chr2:26683517-26683615	OTOF
38	chr2:26683722-26683898	OTOF
39	chr2:26684566-26684805	OTOF
40	chr2:26684953-26685049	OTOF
41	chr2:26686347-26686445	OTOF

42	chr2:26686834-26686974	OTOF
43	chr2:26687739-26687897	OTOF
44	chr2:26688542-26688710	OTOF
45	chr2:26688819-26688944	OTOF
46	chr2:26689584-26689719	OTOF
47	chr2:26689969-26690101	OTOF
48	chr2:26690235-26690369	OTOF
49	chr2:26691262-26691359	OTOF
50	chr2:26693463-26693589	OTOF
51	chr2:26693956-26694053	OTOF
52	chr2:26695385-26695517	OTOF
53	chr2:26696002-26696162	OTOF
54	chr2:26696276-26696435	OTOF
55	chr2:26696861-26696978	OTOF
56	chr2:26697383-26697542	OTOF
57	chr2:26698229-26698361	OTOF
58	chr2:26698784-26698906	OTOF
59	chr2:26698998-26699185	OTOF
60	chr2:26699761-26699911	OTOF
61	chr2:26700042-26700156	OTOF
62	chr2:26700281-26700379	OTOF
63	chr2:26700516-26700761	OTOF
64	chr2:26702134-26702252	OTOF
65	chr2:26702343-26702521	OTOF
66	chr2:26703073-26703179	OTOF
67	chr2:26703656-26703877	OTOF
68	chr2:26705276-26705460	OTOF
69	chr2:26706332-26706516	OTOF
70	chr2:26707344-26707501	OTOF
71	chr2:26712073-26712171	OTOF
72	chr2:26712530-26712627	OTOF
73	chr2:26717812-26717941	OTOF
74	chr2:26724602-26724699	OTOF
75	chr2:26725170-26725294	OTOF
76	chr2:26726629-26726726	OTOF
77	chr2:26739288-26739467	OTOF
78	chr2:26741880-26741977	OTOF
79	chr2:26750696-26750794	OTOF
80	chr2:26760566-26760663	OTOF
81	chr2:26781353-26781450	OTOF
82	chr2:71163087-71163202	ATP6V1B1
83	chr2:71170768-71170865	ATP6V1B1
84	chr2:71185178-71185274	ATP6V1B1
85	chr2:71185462-71185559	ATP6V1B1
86	chr2:71186139-71186236	ATP6V1B1

87	chr2:71187071-71187208	ATP6V1B1
88	chr2:71188053-71188152	ATP6V1B1
89	chr2:71188727-71188824	ATP6V1B1
90	chr2:71189909-71190030	ATP6V1B1
91	chr2:71190294-71190442	ATP6V1B1
92	chr2:71190695-71190793	ATP6V1B1
93	chr2:71191570-71191672	ATP6V1B1
94	chr2:71191868-71191995	ATP6V1B1
95	chr2:71192090-71192251	ATP6V1B1
96	chr2:128015174-128015303	ERCC3
97	chr2:128016874-128017024	ERCC3
98	chr2:128018806-128018922	ERCC3
99	chr2:128028914-128029029	ERCC3
100	chr2:128030441-128030539	ERCC3
101	chr2:128036751-128036951	ERCC3
102	chr2:128038025-128038207	ERCC3
103	chr2:128044281-128044593	ERCC3
104	chr2:128046238-128046440	ERCC3
105	chr2:128046915-128047077	ERCC3
106	chr2:128047267-128047400	ERCC3
107	chr2:128047777-128047874	ERCC3
108	chr2:128050188-128050422	ERCC3
109	chr2:128051091-128051294	ERCC3
110	chr2:128051596-128051693	ERCC3
111	chr2:179318139-179318347	DFNB59
112	chr2:179318559-179325010	DFNB59
113	chr2:179325077-179325173	DFNB59
114	chr2:179325711-179326001	DFNB59
115	chr2:179326076-179326173	DFNB59
116	chr2:219525713-219526030	BCS1L
117	chr2:219526131-219526268	BCS1L
118	chr2:219526484-219526676	BCS1L
119	chr2:219526904-219527001	BCS1L
120	chr2:219527235-219527402	BCS1L
121	chr2:219527608-219527723	BCS1L
122	chr2:219527859-219528109	BCS1L
123	chr2:223065889-223065989	PAX3
124	chr2:223066097-223066194	PAX3
125	chr2:223066645-223066909	PAX3
126	chr2:223084861-223085073	PAX3
127	chr2:223085943-223086106	PAX3
128	chr2:223096799-223097002	PAX3
129	chr2:223158412-223158509	PAX3
130	chr2:223158826-223159020	PAX3
131	chr2:223160249-223160376	PAX3

132	chr2:223161699-223161932	PAX3
133	chr2:223163244-223163342	PAX3
134	chr3:46742976-46743074	TMIE
135	chr3:46747282-46747397	TMIE
136	chr3:46750618-46750765	TMIE
137	chr3:46751071-46751178	TMIE
138	chr3:181430151-181431102	SOX2
139	chr3:191047441-191047538	CCDC50
140	chr3:191074861-191074958	CCDC50
141	chr3:191075789-191075913	CCDC50
142	chr3:191078855-191078953	CCDC50
143	chr3:191087710-191087825	CCDC50
144	chr3:191092853-191093378	CCDC50
145	chr3:191097950-191098063	CCDC50
146	chr3:191098451-191098548	CCDC50
147	chr3:191098619-191098721	CCDC50
148	chr3:191100527-191100624	CCDC50
149	chr3:191107287-191107391	CCDC50
150	chr3:191109492-191109589	CCDC50
151	chr4:6279185-6279414	WFS1
152	chr4:6288813-6288911	WFS1
153	chr4:6290716-6290858	WFS1
154	chr4:6292926-6293094	WFS1
155	chr4:6293636-6293734	WFS1
156	chr4:6296770-6296916	WFS1
157	chr4:6302386-6304195	WFS1
158	chr4:88532038-88532136	DSPP
159	chr4:88533251-88533348	DSPP
160	chr4:88533476-88534460	DSPP
161	chr4:88534939-88537720	DSPP
162	chr5:68715215-68716358	MARVELD2
163	chr5:68720398-68720495	MARVELD2
164	chr5:68728356-68728502	MARVELD2
165	chr5:68728751-68728920	MARVELD2
166	chr5:68736255-68736352	MARVELD2
167	chr5:68737361-68737481	MARVELD2
168	chr5:92920732-92921192	NR2F1
169	chr5:92923625-92924150	NR2F1
170	chr5:92929270-92929548	NR2F1
171	chr5:140896420-140896575	DIAPH1
172	chr5:140903705-140903803	DIAPH1
173	chr5:140905607-140905740	DIAPH1
174	chr5:140905866-140906028	DIAPH1
175	chr5:140907142-140907264	DIAPH1
176	chr5:140908022-140908149	DIAPH1

177	chr5:140908271-140908508	DIAPH1
178	chr5:140908741-140908840	DIAPH1
179	chr5:140909169-140909267	DIAPH1
180	chr5:140913904-140914000	DIAPH1
181	chr5:140915578-140915675	DIAPH1
182	chr5:140950951-140951049	DIAPH1
183	chr5:140951495-140951607	DIAPH1
184	chr5:140953061-140953775	DIAPH1
185	chr5:140954536-140954713	DIAPH1
186	chr5:140955781-140955879	DIAPH1
187	chr5:140956325-140956438	DIAPH1
188	chr5:140957044-140957158	DIAPH1
189	chr5:140957794-140957910	DIAPH1
190	chr5:140958084-140958192	DIAPH1
191	chr5:140958657-140958763	DIAPH1
192	chr5:140960313-140960450	DIAPH1
193	chr5:140961863-140961960	DIAPH1
194	chr5:140962768-140962866	DIAPH1
195	chr5:140963054-140963182	DIAPH1
196	chr5:140963693-140963792	DIAPH1
197	chr5:140966611-140966764	DIAPH1
198	chr5:140967756-140967854	DIAPH1
199	chr5:140998367-140998481	DIAPH1
200	chr5:145718678-145718795	POU4F3
201	chr5:145719113-145720007	POU4F3
202	chr5:147443587-147443685	SPINK5
203	chr5:147444875-147444972	SPINK5
204	chr5:147449888-147450013	SPINK5
205	chr5:147451699-147451797	SPINK5
206	chr5:147465970-147466095	SPINK5
207	chr5:147468089-147468186	SPINK5
208	chr5:147469059-147469184	SPINK5
209	chr5:147470712-147470809	SPINK5
210	chr5:147473919-147474044	SPINK5
211	chr5:147475377-147475474	SPINK5
212	chr5:147477432-147477557	SPINK5
213	chr5:147478790-147478887	SPINK5
214	chr5:147480019-147480144	SPINK5
215	chr5:147480911-147481008	SPINK5
216	chr5:147481346-147481471	SPINK5
217	chr5:147484491-147484589	SPINK5
218	chr5:147486602-147486727	SPINK5
219	chr5:147488310-147488408	SPINK5
220	chr5:147491333-147491458	SPINK5
221	chr5:147492416-147492514	SPINK5

222	chr5:147493927-147494052	SPINK5
223	chr5:147495933-147496031	SPINK5
224	chr5:147498002-147498127	SPINK5
225	chr5:147498537-147498635	SPINK5
226	chr5:147499574-147499699	SPINK5
227	chr5:147499858-147499956	SPINK5
228	chr5:147503398-147503523	SPINK5
229	chr5:147504322-147504420	SPINK5
230	chr5:147505084-147505181	SPINK5
231	chr5:147505288-147505413	SPINK5
232	chr5:147506546-147506644	SPINK5
233	chr5:147510824-147510952	SPINK5
234	chr5:147513357-147513455	SPINK5
235	chr5:147516502-147516600	SPINK5
236	chr6:33132631-33132741	COL11A2
237	chr6:33133328-33133593	COL11A2
238	chr6:33133687-33133784	COL11A2
239	chr6:33133918-33134015	COL11A2
240	chr6:33134269-33134366	COL11A2
241	chr6:33134826-33134923	COL11A2
242	chr6:33136462-33136559	COL11A2
243	chr6:33137587-33137684	COL11A2
244	chr6:33138082-33138179	COL11A2
245	chr6:33138870-33138967	COL11A2
246	chr6:33139027-33139124	COL11A2
247	chr6:33139246-33139351	COL11A2
248	chr6:33141258-33141355	COL11A2
249	chr6:33141629-33141726	COL11A2
250	chr6:33144473-33144570	COL11A2
251	chr6:33146829-33146926	COL11A2
252	chr6:33147491-33147589	COL11A2
253	chr6:33148714-33148811	COL11A2
254	chr6:33148870-33148967	COL11A2
255	chr6:33151925-33152101	COL11A2
256	chr6:33152753-33152850	COL11A2
257	chr6:33154332-33154595	COL11A2
258	chr6:33156757-33156965	COL11A2
259	chr6:35773450-35773859	LHFPL5
260	chr6:35782325-35782559	LHFPL5
261	chr6:35787172-35787269	LHFPL5
262	chr6:76527267-76527381	MYO6
263	chr6:76532486-76532583	MYO6
264	chr6:76538246-76538343	MYO6
265	chr6:76540135-76540262	MYO6
266	chr6:76542561-76542664	MYO6

267	chr6:76545598-76545695	MYO6
268	chr6:76550303-76550400	MYO6
269	chr6:76550933-76551095	MYO6
270	chr6:76554606-76554704	MYO6
271	chr6:76558070-76558248	MYO6
272	chr6:76564858-76565000	MYO6
273	chr6:76566816-76566971	MYO6
274	chr6:76568617-76568714	MYO6
275	chr6:76570729-76570826	MYO6
276	chr6:76572315-76572440	MYO6
277	chr6:76576243-76576340	MYO6
278	chr6:76576651-76576822	MYO6
279	chr6:76580336-76580433	MYO6
280	chr6:76582923-76583020	MYO6
281	chr6:76589539-76589667	MYO6
282	chr6:76589751-76589848	MYO6
283	chr6:76591408-76591535	MYO6
284	chr6:76595718-76595816	MYO6
285	chr6:76596563-76596711	MYO6
286	chr6:76599776-76599982	MYO6
287	chr6:76600937-76601034	MYO6
288	chr6:76602249-76602407	MYO6
289	chr6:76604915-76605012	MYO6
290	chr6:76608062-76608159	MYO6
291	chr6:76617324-76617425	MYO6
292	chr6:76618215-76618344	MYO6
293	chr6:76621355-76621452	MYO6
294	chr6:76623782-76623998	MYO6
295	chr6:76624532-76624729	MYO6
296	chr6:121767996-121769142	GJA1
297	chr6:133595888-133595985	EYA4
298	chr6:133703507-133703604	EYA4
299	chr6:133767770-133767892	EYA4
300	chr6:133769236-133769333	EYA4
301	chr6:133777692-133777790	EYA4
302	chr6:133782238-133782335	EYA4
303	chr6:133783475-133783615	EYA4
304	chr6:133783761-133783902	EYA4
305	chr6:133785909-133786006	EYA4
306	chr6:133789706-133789869	EYA4
307	chr6:133802603-133802737	EYA4
308	chr6:133804164-133804261	EYA4
309	chr6:133827241-133827338	EYA4
310	chr6:133833841-133833938	EYA4
311	chr6:133834018-133834176	EYA4

312	chr6:133836461-133836573	EYA4
313	chr6:133844196-133844315	EYA4
314	chr6:133846155-133846253	EYA4
315	chr6:133846294-133846392	EYA4
316	chr6:133849855-133849953	EYA4
317	chr6:134210538-134210985	TCF21
318	chr6:134212848-134212945	TCF21
319	chr7:5567381-5567522	ACTB
320	chr7:5567637-5567816	ACTB
321	chr7:5567914-5568350	ACTB
322	chr7:5568794-5569031	ACTB
323	chr7:5569168-5569288	ACTB
324	chr7:24738647-24738878	DFNA5
325	chr7:24742368-24742465	DFNA5
326	chr7:24745805-24745995	DFNA5
327	chr7:24747748-24747873	DFNA5
328	chr7:24749845-24750007	DFNA5
329	chr7:24756875-24756993	DFNA5
330	chr7:24758668-24758837	DFNA5
331	chr7:24784183-24784373	DFNA5
332	chr7:24789185-24789393	DFNA5
333	chr7:102993308-102993405	SLC26A5
334	chr7:103014848-103015039	SLC26A5
335	chr7:103017235-103017332	SLC26A5
336	chr7:103018048-103018246	SLC26A5
337	chr7:103018895-103019000	SLC26A5
338	chr7:103019688-103019786	SLC26A5
339	chr7:103020914-103021011	SLC26A5
340	chr7:103029457-103029561	SLC26A5
341	chr7:103029776-103029873	SLC26A5
342	chr7:103030867-103030964	SLC26A5
343	chr7:103032071-103032182	SLC26A5
344	chr7:103033368-103033513	SLC26A5
345	chr7:103038372-103038470	SLC26A5
346	chr7:103048300-103048450	SLC26A5
347	chr7:103050834-103050996	SLC26A5
348	chr7:103051869-103052033	SLC26A5
349	chr7:103053451-103053559	SLC26A5
350	chr7:103061187-103061324	SLC26A5
351	chr7:103061812-103061961	SLC26A5
352	chr7:107302089-107302250	SLC26A4
353	chr7:107303743-107303880	SLC26A4
354	chr7:107312585-107312693	SLC26A4
355	chr7:107314611-107314793	SLC26A4
356	chr7:107315392-107315554	SLC26A4

357	chr7:107323649-107323799	SLC26A4
358	chr7:107323893-107323991	SLC26A4
359	chr7:107329500-107329645	SLC26A4
360	chr7:107330571-107330682	SLC26A4
361	chr7:107334839-107334936	SLC26A4
362	chr7:107335066-107335163	SLC26A4
363	chr7:107336380-107336484	SLC26A4
364	chr7:107338474-107338571	SLC26A4
365	chr7:107340526-107340624	SLC26A4
366	chr7:107341546-107341643	SLC26A4
367	chr7:107342274-107342502	SLC26A4
368	chr7:107344756-107344853	SLC26A4
369	chr7:107350501-107350644	SLC26A4
370	chr7:107352978-107353075	SLC26A4
371	chr7:107355832-107355929	SLC26A4
372	chr8:102504960-102505057	GRHL2
373	chr8:102555471-102555664	GRHL2
374	chr8:102564929-102565026	GRHL2
375	chr8:102570649-102571040	GRHL2
376	chr8:102582547-102582644	GRHL2
377	chr8:102585898-102586052	GRHL2
378	chr8:102589638-102589747	GRHL2
379	chr8:102611284-102611382	GRHL2
380	chr8:102631769-102631925	GRHL2
381	chr8:102643861-102643958	GRHL2
382	chr8:102644456-102644593	GRHL2
383	chr8:102649093-102649190	GRHL2
384	chr8:102656358-102656456	GRHL2
385	chr8:102661637-102661734	GRHL2
386	chr8:102676667-102676764	GRHL2
387	chr8:102678819-102678931	GRHL2
388	chr9:21802717-21802814	MTAP
389	chr9:21815427-21815525	MTAP
390	chr9:21816695-21816792	MTAP
391	chr9:21818036-21818201	MTAP
392	chr9:21837909-21838009	MTAP
393	chr9:21854632-21854869	MTAP
394	chr9:21859304-21859424	MTAP
395	chr9:21861947-21862044	MTAP
396	chr9:75263525-75263622	TMC1
397	chr9:75303601-75303698	TMC1
398	chr9:75309461-75309630	TMC1
399	chr9:75315436-75315559	TMC1
400	chr9:75355032-75355130	TMC1
401	chr9:75357353-75357450	TMC1

402	chr9:75366768-75366872	TMC1
403	chr9:75369704-75369800	TMC1
404	chr9:75387331-75387471	TMC1
405	chr9:75403257-75403399	TMC1
406	chr9:75404041-75404233	TMC1
407	chr9:75406804-75406981	TMC1
408	chr9:75407109-75407268	TMC1
409	chr9:75420300-75420426	TMC1
410	chr9:75431045-75431142	TMC1
411	chr9:75435760-75435997	TMC1
412	chr9:75441787-75441910	TMC1
413	chr9:75445359-75445456	TMC1
414	chr9:75445525-75445622	TMC1
415	chr9:75450831-75450928	TMC1
416	chr9:117165036-117165216	DFNB31
417	chr9:117165499-117165619	DFNB31
418	chr9:117166178-117166357	DFNB31
419	chr9:117168637-117169172	DFNB31
420	chr9:117170215-117170312	DFNB31
421	chr9:117185596-117185803	DFNB31
422	chr9:117186616-117186826	DFNB31
423	chr9:117187239-117187336	DFNB31
424	chr9:117188493-117188693	DFNB31
425	chr9:117228549-117228672	DFNB31
426	chr9:117240835-117241051	DFNB31
427	chr9:117266466-117267081	DFNB31
428	chr9:139089173-139089589	LHX3
429	chr9:139090500-139090666	LHX3
430	chr9:139090756-139090905	LHX3
431	chr9:139091526-139091726	LHX3
432	chr9:139092430-139092599	LHX3
433	chr9:139094791-139094888	LHX3
434	chr9:139096772-139096869	LHX3
435	chr10:8097621-8097859	GATA3
436	chr10:8100270-8100804	GATA3
437	chr10:8105958-8106101	GATA3
438	chr10:8111438-8111561	GATA3
439	chr10:8115704-8115986	GATA3
440	chr10:26241042-26241207	MYO3A
441	chr10:26243805-26243937	MYO3A
442	chr10:26285421-26285523	MYO3A
443	chr10:26286090-26286187	MYO3A
444	chr10:26305740-26305837	MYO3A
445	chr10:26310434-26310577	MYO3A
446	chr10:26312936-26313033	MYO3A

447	chr10:26315308-26315461	MYO3A
448	chr10:26355906-26356003	MYO3A
449	chr10:26357699-26357813	MYO3A
450	chr10:26359042-26359144	MYO3A
451	chr10:26359241-26359338	MYO3A
452	chr10:26377134-26377334	MYO3A
453	chr10:26385312-26385408	MYO3A
454	chr10:26385499-26385611	MYO3A
455	chr10:26409607-26409731	MYO3A
456	chr10:26414329-26414537	MYO3A
457	chr10:26417322-26417467	MYO3A
458	chr10:26432379-26432530	MYO3A
459	chr10:26434371-26434469	MYO3A
460	chr10:26436361-26436488	MYO3A
461	chr10:26442771-26442868	MYO3A
462	chr10:26443666-26443763	MYO3A
463	chr10:26446241-26446444	MYO3A
464	chr10:26454998-26455107	MYO3A
465	chr10:26457643-26457803	MYO3A
466	chr10:26459347-26459468	MYO3A
467	chr10:26462594-26463486	MYO3A
468	chr10:26465632-26465774	MYO3A
469	chr10:26482136-26482240	MYO3A
470	chr10:26490167-26490264	MYO3A
471	chr10:26491895-26492036	MYO3A
472	chr10:26500774-26500892	MYO3A
473	chr10:55566342-55566890	PCDH15
474	chr10:55568456-55569306	PCDH15
475	chr10:55570318-55570426	PCDH15
476	chr10:55571297-55571395	PCDH15
477	chr10:55581620-55583118	PCDH15
478	chr10:55583144-55583241	PCDH15
479	chr10:55584850-55584948	PCDH15
480	chr10:55587152-55587308	PCDH15
481	chr10:55588282-55588379	PCDH15
482	chr10:55591077-55591293	PCDH15
483	chr10:55600082-55600256	PCDH15
484	chr10:55616931-55617029	PCDH15
485	chr10:55626404-55626617	PCDH15
486	chr10:55663005-55663130	PCDH15
487	chr10:55698577-55698715	PCDH15
488	chr10:55700628-55700735	PCDH15
489	chr10:55719494-55719604	PCDH15
490	chr10:55721514-55721652	PCDH15
491	chr10:55755411-55755525	PCDH15

492	chr10:55779954-55780176	PCDH15
493	chr10:55782654-55782957	PCDH15
494	chr10:55826519-55826645	PCDH15
495	chr10:55839090-55839187	PCDH15
496	chr10:55849736-55849833	PCDH15
497	chr10:55892637-55892767	PCDH15
498	chr10:55912862-55913053	PCDH15
499	chr10:55943206-55943353	PCDH15
500	chr10:55944896-55945028	PCDH15
501	chr10:55949087-55949184	PCDH15
502	chr10:55955445-55955649	PCDH15
503	chr10:55973698-55973808	PCDH15
504	chr10:55996585-55996691	PCDH15
505	chr10:56077033-56077201	PCDH15
506	chr10:56089358-56089466	PCDH15
507	chr10:56106127-56106244	PCDH15
508	chr10:56128882-56129035	PCDH15
509	chr10:56138544-56138702	PCDH15
510	chr10:56287557-56287654	PCDH15
511	chr10:56288108-56288205	PCDH15
512	chr10:56367628-56367726	PCDH15
513	chr10:56423929-56424027	PCDH15
514	chr10:73199574-73199672	CDH23
515	chr10:73206066-73206163	CDH23
516	chr10:73269841-73269981	CDH23
517	chr10:73270687-73270784	CDH23
518	chr10:73270875-73270973	CDH23
519	chr10:73326501-73326693	CDH23
520	chr10:73330549-73330675	CDH23
521	chr10:73337662-73337760	CDH23
522	chr10:73375263-73375373	CDH23
523	chr10:73376964-73377150	CDH23
524	chr10:73403573-73403670	CDH23
525	chr10:73405585-73405737	CDH23
526	chr10:73406218-73406518	CDH23
527	chr10:73434854-73434951	CDH23
528	chr10:73437216-73437450	CDH23
529	chr10:73439147-73439249	CDH23
530	chr10:73442205-73442329	CDH23
531	chr10:73447393-73447490	CDH23
532	chr10:73450228-73450341	CDH23
533	chr10:73453907-73454016	CDH23
534	chr10:73455178-73455282	CDH23
535	chr10:73461782-73461968	CDH23
536	chr10:73462309-73462451	CDH23

537	chr10:73464671-73464887	CDH23
538	chr10:73466657-73466806	CDH23
539	chr10:73468858-73468968	CDH23
540	chr10:73472425-73472570	CDH23
541	chr10:73483785-73483882	CDH23
542	chr10:73485132-73485277	CDH23
543	chr10:73490229-73490361	CDH23
544	chr10:73491747-73492132	CDH23
545	chr10:73494000-73494098	CDH23
546	chr10:73498255-73498404	CDH23
547	chr10:73499404-73499529	CDH23
548	chr10:73500582-73500707	CDH23
549	chr10:73501454-73501678	CDH23
550	chr10:73537440-73537658	CDH23
551	chr10:73537949-73538065	CDH23
552	chr10:73539027-73539204	CDH23
553	chr10:73544047-73544177	CDH23
554	chr10:73544651-73544857	CDH23
555	chr10:73545391-73545495	CDH23
556	chr10:73548700-73548799	CDH23
557	chr10:73550048-73550170	CDH23
558	chr10:73550892-73551092	CDH23
559	chr10:73552942-73553397	CDH23
560	chr10:73556864-73556977	CDH23
561	chr10:73558114-73558335	CDH23
562	chr10:73558871-73559037	CDH23
563	chr10:73559252-73559386	CDH23
564	chr10:73560396-73560512	CDH23
565	chr10:73562658-73562832	CDH23
566	chr10:73562969-73563177	CDH23
567	chr10:73565566-73565754	CDH23
568	chr10:73565928-73566038	CDH23
569	chr10:73567037-73567163	CDH23
570	chr10:73567276-73567524	CDH23
571	chr10:73567606-73567764	CDH23
572	chr10:73569580-73569833	CDH23
573	chr10:73570230-73570328	CDH23
574	chr10:73571075-73571192	CDH23
575	chr10:73571260-73571358	CDH23
576	chr10:73571443-73571540	CDH23
577	chr10:73571695-73571792	CDH23
578	chr10:73572240-73572366	CDH23
579	chr10:73572528-73572647	CDH23
580	chr10:73573004-73573105	CDH23
581	chr10:73574712-73575035	CDH23

582	chr10:102777858-102778053	PDZD7
583	chr10:102778581-102778974	PDZD7
584	chr10:102780358-102780455	PDZD7
585	chr10:102781557-102781702	PDZD7
586	chr10:102781968-102782142	PDZD7
587	chr10:102783195-102783367	PDZD7
588	chr10:102783687-102783825	PDZD7
589	chr10:102789753-102789976	PDZD7
590	chr11:17515862-17515959	USH1C
591	chr11:17517118-17517224	USH1C
592	chr11:17518285-17518382	USH1C
593	chr11:17519711-17519818	USH1C
594	chr11:17522600-17522697	USH1C
595	chr11:17523008-17523105	USH1C
596	chr11:17523459-17523556	USH1C
597	chr11:17526172-17526269	USH1C
598	chr11:17527379-17527496	USH1C
599	chr11:17530905-17531385	USH1C
600	chr11:17531954-17532068	USH1C
601	chr11:17533451-17533601	USH1C
602	chr11:17537765-17537862	USH1C
603	chr11:17538937-17539034	USH1C
604	chr11:17542419-17542541	USH1C
605	chr11:17542878-17542975	USH1C
606	chr11:17544333-17544473	USH1C
607	chr11:17544739-17544836	USH1C
608	chr11:17544948-17545045	USH1C
609	chr11:17545992-17546090	USH1C
610	chr11:17547893-17547991	USH1C
611	chr11:17548281-17548378	USH1C
612	chr11:17548528-17548625	USH1C
613	chr11:17548772-17548878	USH1C
614	chr11:17552703-17552839	USH1C
615	chr11:17552948-17553089	USH1C
616	chr11:17554788-17554885	USH1C
617	chr11:17565789-17565886	USH1C
618	chr11:67351267-67351365	GSTP1
619	chr11:67351575-67351672	GSTP1
620	chr11:67351937-67352041	GSTP1
621	chr11:67352152-67352249	GSTP1
622	chr11:67352611-67352712	GSTP1
623	chr11:67353577-67353682	GSTP1
624	chr11:67353862-67354048	GSTP1
625	chr11:69625075-69625468	FGF3
626	chr11:69631090-69631191	FGF3

627	chr11:69633484-69633701	FGF3
628	chr11:71800123-71800220	LRTOMT
629	chr11:71804544-71804747	LRTOMT
630	chr11:71805996-71806142	LRTOMT
631	chr11:71806427-71806566	LRTOMT
632	chr11:71807741-71807838	LRTOMT
633	chr11:71815986-71816084	LRTOMT
634	chr11:71816984-71817256	LRTOMT
635	chr11:71818971-71819165	LRTOMT
636	chr11:71819653-71819971	LRTOMT
637	chr11:76841642-76841740	MYO7A
638	chr11:76853758-76853868	MYO7A
639	chr11:76858847-76858996	MYO7A
640	chr11:76866956-76867137	MYO7A
641	chr11:76867709-76867827	MYO7A
642	chr11:76867911-76868050	MYO7A
643	chr11:76868328-76868438	MYO7A
644	chr11:76869326-76869476	MYO7A
645	chr11:76870484-76870581	MYO7A
646	chr11:76871212-76871328	MYO7A
647	chr11:76872022-76872161	MYO7A
648	chr11:76873169-76873376	MYO7A
649	chr11:76873902-76874034	MYO7A
650	chr11:76877105-76877208	MYO7A
651	chr11:76883797-76883931	MYO7A
652	chr11:76885805-76885960	MYO7A
653	chr11:76886417-76886514	MYO7A
654	chr11:76888595-76888692	MYO7A
655	chr11:76890086-76890183	MYO7A
656	chr11:76890784-76890999	MYO7A
657	chr11:76891423-76891527	MYO7A
658	chr11:76892429-76892635	MYO7A
659	chr11:76893000-76893200	MYO7A
660	chr11:76893472-76893645	MYO7A
661	chr11:76894110-76894208	MYO7A
662	chr11:76895636-76895794	MYO7A
663	chr11:76900392-76900515	MYO7A
664	chr11:76901068-76901184	MYO7A
665	chr11:76901745-76901915	MYO7A
666	chr11:76903099-76903323	MYO7A
667	chr11:76905402-76905569	MYO7A
668	chr11:76908529-76908643	MYO7A
669	chr11:76909543-76909666	MYO7A
670	chr11:76910583-76910863	MYO7A
671	chr11:76912496-76912683	MYO7A

672	chr11:76913348-76913469	MYO7A
673	chr11:76914108-76914262	MYO7A
674	chr11:76915123-76915274	MYO7A
675	chr11:76916510-76916662	MYO7A
676	chr11:76917145-76917247	MYO7A
677	chr11:76918337-76918447	MYO7A
678	chr11:76919471-76919569	MYO7A
679	chr11:76919745-76919848	MYO7A
680	chr11:76922200-76922382	MYO7A
681	chr11:76922869-76922982	MYO7A
682	chr11:76923991-76924089	MYO7A
683	chr11:76924908-76925024	MYO7A
684	chr11:76925649-76925747	MYO7A
685	chr11:110102596-110102758	RDX
686	chr11:110103964-110104204	RDX
687	chr11:110106822-110106920	RDX
688	chr11:110108219-110108377	RDX
689	chr11:110118430-110118558	RDX
690	chr11:110124673-110124834	RDX
691	chr11:110126014-110126112	RDX
692	chr11:110128494-110128638	RDX
693	chr11:110128825-110128922	RDX
694	chr11:110134687-110134959	RDX
695	chr11:110135504-110135601	RDX
696	chr11:110143255-110143352	RDX
697	chr11:110150364-110150461	RDX
698	chr11:113558941-113559039	TMPRSS5
699	chr11:113560489-113560639	TMPRSS5
700	chr11:113560955-113561095	TMPRSS5
701	chr11:113561565-113561661	TMPRSS5
702	chr11:113563795-113563971	TMPRSS5
703	chr11:113565202-113565362	TMPRSS5
704	chr11:113566096-113566193	TMPRSS5
705	chr11:113567582-113567693	TMPRSS5
706	chr11:113568007-113568140	TMPRSS5
707	chr11:113569629-113569749	TMPRSS5
708	chr11:113570319-113570415	TMPRSS5
709	chr11:113570790-113570890	TMPRSS5
710	chr11:113576897-113576995	TMPRSS5
711	chr11:120973359-120973456	TECTA
712	chr11:120976542-120976673	TECTA
713	chr11:120979922-120980207	TECTA
714	chr11:120983783-120983918	TECTA
715	chr11:120984264-120984427	TECTA
716	chr11:120989017-120989427	TECTA

717	chr11:120996013-120996581	TECTA
718	chr11:120998463-120999053	TECTA
719	chr11:121000349-121000920	TECTA
720	chr11:121008132-121008731	TECTA
721	chr11:121016266-121016825	TECTA
722	chr11:121023592-121023789	TECTA
723	chr11:121028552-121028933	TECTA
724	chr11:121030846-121031130	TECTA
725	chr11:121032786-121033079	TECTA
726	chr11:121035984-121036092	TECTA
727	chr11:121037289-121037489	TECTA
728	chr11:121038765-121038926	TECTA
729	chr11:121039388-121039634	TECTA
730	chr11:121058543-121058703	TECTA
731	chr11:121059785-121059882	TECTA
732	chr11:121060475-121060589	TECTA
733	chr11:121061417-121061515	TECTA
734	chr12:57422541-57422665	MYO1A
735	chr12:57422918-57423043	MYO1A
736	chr12:57423221-57423371	MYO1A
737	chr12:57423526-57423656	MYO1A
738	chr12:57423996-57424100	MYO1A
739	chr12:57424826-57424958	MYO1A
740	chr12:57430081-57430178	MYO1A
741	chr12:57430543-57430640	MYO1A
742	chr12:57430728-57430875	MYO1A
743	chr12:57431331-57431428	MYO1A
744	chr12:57431655-57431853	MYO1A
745	chr12:57432198-57432422	MYO1A
746	chr12:57432595-57432793	MYO1A
747	chr12:57432980-57433077	MYO1A
748	chr12:57434970-57435072	MYO1A
749	chr12:57435201-57435298	MYO1A
750	chr12:57436851-57436949	MYO1A
751	chr12:57437026-57437142	MYO1A
752	chr12:57437642-57437787	MYO1A
753	chr12:57437892-57437993	MYO1A
754	chr12:57440338-57440434	MYO1A
755	chr12:57440631-57440728	MYO1A
756	chr12:57440821-57440918	MYO1A
757	chr12:57441089-57441191	MYO1A
758	chr12:57441410-57441508	MYO1A
759	chr12:57441775-57441888	MYO1A
760	chr12:57441996-57442107	MYO1A
761	chr12:100751172-100751270	SLC17A8

762	chr12:100774481-100774731	SLC17A8
763	chr12:100784781-100784897	SLC17A8
764	chr12:100787149-100787261	SLC17A8
765	chr12:100790104-100790201	SLC17A8
766	chr12:100795550-100795648	SLC17A8
767	chr12:100796120-100796257	SLC17A8
768	chr12:100796376-100796523	SLC17A8
769	chr12:100797818-100797948	SLC17A8
770	chr12:100806550-100806658	SLC17A8
771	chr12:100811809-100811934	SLC17A8
772	chr12:100813595-100813937	SLC17A8
773	chr13:20763042-20763720	GJB2
774	chr13:20796836-20797619	GJB6
775	chr14:31344114-31344211	COCH
776	chr14:31344238-31344335	COCH
777	chr14:31346780-31346934	COCH
778	chr14:31348019-31348150	COCH
779	chr14:31348613-31348710	COCH
780	chr14:31349633-31349730	COCH
781	chr14:31349795-31349940	COCH
782	chr14:31353761-31353862	COCH
783	chr14:31354602-31354826	COCH
784	chr14:31355004-31355518	COCH
785	chr14:31358824-31358997	COCH
786	chr14:76905699-76906093	ESRRB
787	chr14:76928890-76929004	ESRRB
788	chr14:76948361-76948469	ESRRB
789	chr14:76948943-76949102	ESRRB
790	chr14:76957792-76958059	ESRRB
791	chr14:76964559-76964795	ESRRB
792	chr14:76965071-76965255	ESRRB
793	chr14:76966178-76966409	ESRRB
794	chr14:76967004-76967101	ESRRB
795	chr15:43891866-43891964	STRC
796	chr15:43892160-43892305	STRC
797	chr15:43892395-43892492	STRC
798	chr15:43892734-43892880	STRC
799	chr15:43893072-43893212	STRC
800	chr15:43893596-43893749	STRC
801	chr15:43895442-43895609	STRC
802	chr15:43896196-43896350	STRC
803	chr15:43896556-43896654	STRC
804	chr15:43896850-43897044	STRC
805	chr15:43897464-43897597	STRC
806	chr15:43900063-43900173	STRC

807	chr15:43900280-43900401	STRC
808	chr15:43901456-43901553	STRC
809	chr15:43902512-43902635	STRC
810	chr15:43903102-43903199	STRC
811	chr15:43903348-43903513	STRC
812	chr15:43903669-43903766	STRC
813	chr15:43904042-43904222	STRC
814	chr15:43904577-43904707	STRC
815	chr15:43904991-43905104	STRC
816	chr15:43905245-43905429	STRC
817	chr15:43906087-43906251	STRC
818	chr15:43906396-43906510	STRC
819	chr15:43906596-43906693	STRC
820	chr15:43907633-43908888	STRC
821	chr15:43909621-43909718	STRC
822	chr15:43909771-43910554	STRC
823	chr15:43910841-43910938	STRC
824	chr15:43922867-43922964	CATSPER2
825	chr15:43924399-43924561	CATSPER2
826	chr15:43924917-43925132	CATSPER2
827	chr15:43927539-43927636	CATSPER2
828	chr15:43927927-43928024	CATSPER2
829	chr15:43928241-43928417	CATSPER2
830	chr15:43931104-43931226	CATSPER2
831	chr15:43931843-43931996	CATSPER2
832	chr15:43932486-43932694	CATSPER2
833	chr15:43939235-43939332	CATSPER2
834	chr15:43939494-43939665	CATSPER2
835	chr15:43940117-43940259	CATSPER2
836	chr16:21270087-21270184	CRYM
837	chr16:21272569-21272667	CRYM
838	chr16:21273360-21273479	CRYM
839	chr16:21278877-21279058	CRYM
840	chr16:21281113-21281212	CRYM
841	chr16:21286837-21286934	CRYM
842	chr16:21288754-21288905	CRYM
843	chr16:21289405-21289572	CRYM
844	chr16:21689833-21689931	OTOA
845	chr16:21690203-21690300	OTOA
846	chr16:21690325-21690422	OTOA
847	chr16:21690471-21690568	OTOA
848	chr16:21693055-21693152	OTOA
849	chr16:21695811-21695909	OTOA
850	chr16:21696553-21696682	OTOA
851	chr16:21698736-21698969	OTOA

852	chr16:21702907-21703008	OTOA
853	chr16:21709098-21709196	OTOA
854	chr16:21712211-21712348	OTOA
855	chr16:21716307-21716404	OTOA
856	chr16:21716492-21716613	OTOA
857	chr16:21721211-21721424	OTOA
858	chr16:21726308-21726473	OTOA
859	chr16:21728230-21728368	OTOA
860	chr16:21730436-21730533	OTOA
861	chr16:21730710-21730825	OTOA
862	chr16:21734215-21734312	OTOA
863	chr16:21737846-21737979	OTOA
864	chr16:21739564-21739752	OTOA
865	chr16:21742157-21742254	OTOA
866	chr16:21747584-21747711	OTOA
867	chr16:21752044-21752229	OTOA
868	chr16:21756204-21756357	OTOA
869	chr16:21763258-21763398	OTOA
870	chr16:21763692-21763826	OTOA
871	chr16:21764349-21764446	OTOA
872	chr16:21768405-21768598	OTOA
873	chr16:21771779-21771876	OTOA
874	chr17:1368986-1369083	MYO1C
875	chr17:1370538-1370635	MYO1C
876	chr17:1370775-1370872	MYO1C
877	chr17:1371122-1371219	MYO1C
878	chr17:1371284-1371417	MYO1C
879	chr17:1371527-1371624	MYO1C
880	chr17:1371696-1371793	MYO1C
881	chr17:1372819-1372916	MYO1C
882	chr17:1373471-1373628	MYO1C
883	chr17:1373723-1373821	MYO1C
884	chr17:1373902-1373999	MYO1C
885	chr17:1374356-1374453	MYO1C
886	chr17:1374519-1374630	MYO1C
887	chr17:1375203-1375318	MYO1C
888	chr17:1375419-1375522	MYO1C
889	chr17:1377896-1377994	MYO1C
890	chr17:1378082-1378179	MYO1C
891	chr17:1378231-1378329	MYO1C
892	chr17:1380797-1380894	MYO1C
893	chr17:1381177-1381275	MYO1C
894	chr17:1381383-1381486	MYO1C
895	chr17:1381697-1381795	MYO1C
896	chr17:1381912-1382029	MYO1C

897	chr17:1382713-1382810	MYO1C
898	chr17:1382888-1382999	MYO1C
899	chr17:1383823-1383919	MYO1C
900	chr17:1384002-1384179	MYO1C
901	chr17:1385764-1385862	MYO1C
902	chr17:1386157-1386353	MYO1C
903	chr17:1386906-1387019	MYO1C
904	chr17:1387444-1387597	MYO1C
905	chr17:1388949-1389046	MYO1C
906	chr17:1395696-1395794	MYO1C
907	chr17:15134236-15134397	PMP22
908	chr17:15142790-15142928	PMP22
909	chr17:15162413-15162510	PMP22
910	chr17:15163958-15164055	PMP22
911	chr17:18022117-18025723	MYO15A
912	chr17:18027790-18027888	MYO15A
913	chr17:18028466-18028563	MYO15A
914	chr17:18029663-18029770	MYO15A
915	chr17:18030094-18030192	MYO15A
916	chr17:18030388-18030486	MYO15A
917	chr17:18034074-18034171	MYO15A
918	chr17:18034554-18034656	MYO15A
919	chr17:18034766-18034863	MYO15A
920	chr17:18035769-18035880	MYO15A
921	chr17:18036541-18036700	MYO15A
922	chr17:18039027-18039138	MYO15A
923	chr17:18039712-18039810	MYO15A
924	chr17:18039879-18040000	MYO15A
925	chr17:18040898-18040995	MYO15A
926	chr17:18041431-18041560	MYO15A
927	chr17:18042127-18042250	MYO15A
928	chr17:18042839-18042936	MYO15A
929	chr17:18043833-18043979	MYO15A
930	chr17:18044076-18044173	MYO15A
931	chr17:18044335-18044457	MYO15A
932	chr17:18044969-18045084	MYO15A
933	chr17:18045395-18045568	MYO15A
934	chr17:18046064-18046162	MYO15A
935	chr17:18046859-18046956	MYO15A
936	chr17:18047022-18047119	MYO15A
937	chr17:18047186-18047314	MYO15A
938	chr17:18047811-18047908	MYO15A
939	chr17:18049188-18049421	MYO15A
940	chr17:18051345-18051524	MYO15A
941	chr17:18051798-18051896	MYO15A

942	chr17:18052077-18052266	MYO15A
943	chr17:18052532-18052690	MYO15A
944	chr17:18052799-18052897	MYO15A
945	chr17:18053745-18053857	MYO15A
946	chr17:18053998-18054095	MYO15A
947	chr17:18054141-18054238	MYO15A
948	chr17:18054426-18054604	MYO15A
949	chr17:18054711-18054841	MYO15A
950	chr17:18055162-18055265	MYO15A
951	chr17:18055414-18055512	MYO15A
952	chr17:18057091-18057210	MYO15A
953	chr17:18057427-18057524	MYO15A
954	chr17:18057984-18058081	MYO15A
955	chr17:18058426-18058539	MYO15A
956	chr17:18058630-18058746	MYO15A
957	chr17:18059511-18059650	MYO15A
958	chr17:18060270-18060379	MYO15A
959	chr17:18060459-18060557	MYO15A
960	chr17:18061038-18061214	MYO15A
961	chr17:18061839-18061952	MYO15A
962	chr17:18062227-18062325	MYO15A
963	chr17:18062577-18062675	MYO15A
964	chr17:18062909-18063006	MYO15A
965	chr17:18063242-18063340	MYO15A
966	chr17:18064633-18064761	MYO15A
967	chr17:18065898-18065996	MYO15A
968	chr17:18066549-18066646	MYO15A
969	chr17:18067056-18067154	MYO15A
970	chr17:18069677-18069835	MYO15A
971	chr17:18070906-18071037	MYO15A
972	chr17:18074954-18075085	MYO15A
973	chr17:18075473-18075604	MYO15A
974	chr17:18077097-18077235	MYO15A
975	chr17:18082085-18082184	MYO15A
976	chr17:79477718-79477859	ACTG1
977	chr17:79477955-79478134	ACTG1
978	chr17:79478216-79478652	ACTG1
979	chr17:79478931-79479168	ACTG1
980	chr17:79479260-79479380	ACTG1
981	chr19:8586402-8586500	MYO1F
982	chr19:8587263-8587430	MYO1F
983	chr19:8587520-8587713	MYO1F
984	chr19:8590357-8590454	MYO1F
985	chr19:8591339-8591485	MYO1F
986	chr19:8591675-8591819	MYO1F

987	chr19:8592224-8592367	MYO1F
988	chr19:8595082-8595249	MYO1F
989	chr19:8595345-8595457	MYO1F
990	chr19:8601138-8601280	MYO1F
991	chr19:8601385-8601481	MYO1F
992	chr19:8601835-8601939	MYO1F
993	chr19:8604824-8604921	MYO1F
994	chr19:8606785-8606882	MYO1F
995	chr19:8609183-8609348	MYO1F
996	chr19:8610529-8610627	MYO1F
997	chr19:8612915-8613013	MYO1F
998	chr19:8613113-8613211	MYO1F
999	chr19:8615046-8615240	MYO1F
1000	chr19:8615448-8615578	MYO1F
1001	chr19:8616626-8616758	MYO1F
1002	chr19:8616919-8617048	MYO1F
1003	chr19:8618020-8618117	MYO1F
1004	chr19:8618230-8618327	MYO1F
1005	chr19:8619360-8619458	MYO1F
1006	chr19:8619535-8619632	MYO1F
1007	chr19:8620545-8620680	MYO1F
1008	chr19:8642144-8642242	MYO1F
1009	chr19:45854885-45854983	ERCC2
1010	chr19:45855469-45855610	ERCC2
1011	chr19:45855766-45855907	ERCC2
1012	chr19:45855992-45856089	ERCC2
1013	chr19:45856330-45856427	ERCC2
1014	chr19:45856498-45856596	ERCC2
1015	chr19:45857990-45858109	ERCC2
1016	chr19:45858907-45859004	ERCC2
1017	chr19:45860530-45860629	ERCC2
1018	chr19:45860719-45860816	ERCC2
1019	chr19:45860875-45860972	ERCC2
1020	chr19:45862096-45862194	ERCC2
1021	chr19:45864784-45864900	ERCC2
1022	chr19:45867003-45867169	ERCC2
1023	chr19:45867246-45867377	ERCC2
1024	chr19:45867493-45867591	ERCC2
1025	chr19:45867684-45867805	ERCC2
1026	chr19:45868098-45868212	ERCC2
1027	chr19:45868302-45868416	ERCC2
1028	chr19:45871890-45872001	ERCC2
1029	chr19:45872172-45872269	ERCC2
1030	chr19:45872319-45872416	ERCC2
1031	chr19:45873393-45873490	ERCC2

1032	chr19:45873749-45873846	ERCC2
1033	chr19:50713625-50714027	MYH14
1034	chr19:50720874-50721028	MYH14
1035	chr19:50726306-50726403	MYH14
1036	chr19:50726506-50726606	MYH14
1037	chr19:50727375-50727472	MYH14
1038	chr19:50728840-50728938	MYH14
1039	chr19:50730144-50730241	MYH14
1040	chr19:50733784-50733880	MYH14
1041	chr19:50735189-50735327	MYH14
1042	chr19:50747499-50747596	MYH14
1043	chr19:50750263-50750379	MYH14
1044	chr19:50752246-50752396	MYH14
1045	chr19:50752909-50753080	MYH14
1046	chr19:50753774-50753945	MYH14
1047	chr19:50755898-50756010	MYH14
1048	chr19:50758479-50758576	MYH14
1049	chr19:50760558-50760743	MYH14
1050	chr19:50762403-50762522	MYH14
1051	chr19:50763890-50763987	MYH14
1052	chr19:50764734-50764892	MYH14
1053	chr19:50766571-50766677	MYH14
1054	chr19:50770136-50770265	MYH14
1055	chr19:50771420-50771624	MYH14
1056	chr19:50774668-50774803	MYH14
1057	chr19:50775108-50775229	MYH14
1058	chr19:50775819-50775988	MYH14
1059	chr19:50779250-50779460	MYH14
1060	chr19:50780016-50780158	MYH14
1061	chr19:50781342-50781546	MYH14
1062	chr19:50783296-50783398	MYH14
1063	chr19:50783490-50783640	MYH14
1064	chr19:50784853-50785099	MYH14
1065	chr19:50789741-50789951	MYH14
1066	chr19:50792695-50792905	MYH14
1067	chr19:50794146-50794305	MYH14
1068	chr19:50795520-50795646	MYH14
1069	chr19:50796486-50796584	MYH14
1070	chr19:50796823-50796944	MYH14
1071	chr19:50804920-50805126	MYH14
1072	chr19:50810308-50810414	MYH14
1073	chr19:50812264-50812434	MYH14
1074	chr19:50812899-50813047	MYH14
1075	chr20:3208420-3208517	SLC4A11
1076	chr20:3208907-3209074	SLC4A11

1077	chr20:3209160-3209353	SLC4A11
1078	chr20:3209486-3209657	SLC4A11
1079	chr20:3209743-3209909	SLC4A11
1080	chr20:3209994-3210098	SLC4A11
1081	chr20:3210172-3210422	SLC4A11
1082	chr20:3210822-3210919	SLC4A11
1083	chr20:3211163-3211293	SLC4A11
1084	chr20:3211380-3211491	SLC4A11
1085	chr20:3211581-3211704	SLC4A11
1086	chr20:3211794-3211891	SLC4A11
1087	chr20:3211978-3212194	SLC4A11
1088	chr20:3214162-3214283	SLC4A11
1089	chr20:3214560-3214657	SLC4A11
1090	chr20:3214731-3214960	SLC4A11
1091	chr20:3215191-3215288	SLC4A11
1092	chr20:3215390-3215540	SLC4A11
1093	chr20:3218192-3218325	SLC4A11
1094	chr20:10620148-10620603	JAG1
1095	chr20:10621433-10621581	JAG1
1096	chr20:10621763-10621892	JAG1
1097	chr20:10622110-10622341	JAG1
1098	chr20:10622433-10622540	JAG1
1099	chr20:10623138-10623249	JAG1
1100	chr20:10624421-10624518	JAG1
1101	chr20:10624971-10625068	JAG1
1102	chr20:10625513-10625627	JAG1
1103	chr20:10625793-10625904	JAG1
1104	chr20:10626006-10626117	JAG1
1105	chr20:10626621-10626732	JAG1
1106	chr20:10627589-10627751	JAG1
1107	chr20:10628610-10628758	JAG1
1108	chr20:10629199-10629370	JAG1
1109	chr20:10629685-10629782	JAG1
1110	chr20:10630172-10630283	JAG1
1111	chr20:10630897-10631008	JAG1
1112	chr20:10632231-10632342	JAG1
1113	chr20:10632781-10632898	JAG1
1114	chr20:10633118-10633246	JAG1
1115	chr20:10637029-10637126	JAG1
1116	chr20:10639118-10639370	JAG1
1117	chr20:10644589-10644686	JAG1
1118	chr20:10653351-10653654	JAG1
1119	chr20:10654090-10654188	JAG1
1120	chr20:16729049-16729161	OTOR
1121	chr20:16729514-16729651	OTOR

1122	chr20:16730550-16730655	OTOR
1123	chr20:16731704-16731801	OTOR
1124	chr20:61448408-61448505	COL9A3
1125	chr20:61448906-61449003	COL9A3
1126	chr20:61449840-61449937	COL9A3
1127	chr20:61450562-61450659	COL9A3
1128	chr20:61451260-61451357	COL9A3
1129	chr20:61452503-61452600	COL9A3
1130	chr20:61452823-61452920	COL9A3
1131	chr20:61453088-61453185	COL9A3
1132	chr20:61453442-61453539	COL9A3
1133	chr20:61453916-61454013	COL9A3
1134	chr20:61455778-61455875	COL9A3
1135	chr20:61456299-61456396	COL9A3
1136	chr20:61457148-61457245	COL9A3
1137	chr20:61457535-61457632	COL9A3
1138	chr20:61458098-61458195	COL9A3
1139	chr20:61458572-61458669	COL9A3
1140	chr20:61459254-61459351	COL9A3
1141	chr20:61460095-61460192	COL9A3
1142	chr20:61460254-61460351	COL9A3
1143	chr20:61460782-61460879	COL9A3
1144	chr20:61460959-61461056	COL9A3
1145	chr20:61461097-61461194	COL9A3
1146	chr20:61461691-61461788	COL9A3
1147	chr20:61461857-61461954	COL9A3
1148	chr20:61463476-61463573	COL9A3
1149	chr20:61464347-61464444	COL9A3
1150	chr20:61467242-61467339	COL9A3
1151	chr20:61467541-61467685	COL9A3
1152	chr20:61467810-61467907	COL9A3
1153	chr20:61468437-61468617	COL9A3
1154	chr20:61470027-61470124	COL9A3
1155	chr20:61471896-61472084	COL9A3
1156	chr21:35821545-35821932	KCNE1
1157	chr21:37833276-37833993	CLDN14
1158	chr21:43792832-43792929	TMPRSS3
1159	chr21:43795827-43795977	TMPRSS3
1160	chr21:43796652-43796795	TMPRSS3
1161	chr21:43800226-43800323	TMPRSS3
1162	chr21:43802093-43802343	TMPRSS3
1163	chr21:43803144-43803307	TMPRSS3
1164	chr21:43804053-43804150	TMPRSS3
1165	chr21:43805520-43805643	TMPRSS3
1166	chr21:43808514-43808635	TMPRSS3

1167	chr21:43809040-43809154	TMPRSS3
1168	chr21:43809550-43809927	TMPRSS3
1169	chr21:43810038-43810146	TMPRSS3
1170	chr21:43815432-43815529	TMPRSS3
1171	chr22:36678716-36678831	MYH9
1172	chr22:36680141-36680311	MYH9
1173	chr22:36680451-36680557	MYH9
1174	chr22:36681169-36681375	MYH9
1175	chr22:36681706-36681827	MYH9
1176	chr22:36681907-36682005	MYH9
1177	chr22:36682766-36682892	MYH9
1178	chr22:36684300-36684459	MYH9
1179	chr22:36684775-36684985	MYH9
1180	chr22:36685133-36685343	MYH9
1181	chr22:36688034-36688280	MYH9
1182	chr22:36689377-36689527	MYH9
1183	chr22:36689807-36689909	MYH9
1184	chr22:36690140-36690344	MYH9
1185	chr22:36690980-36691122	MYH9
1186	chr22:36691553-36691763	MYH9
1187	chr22:36692891-36693060	MYH9
1188	chr22:36694967-36695088	MYH9
1189	chr22:36696175-36696310	MYH9
1190	chr22:36696899-36697103	MYH9
1191	chr22:36697582-36697711	MYH9
1192	chr22:36698616-36698722	MYH9
1193	chr22:36700043-36700201	MYH9
1194	chr22:36701066-36701163	MYH9
1195	chr22:36701978-36702097	MYH9
1196	chr22:36702462-36702653	MYH9
1197	chr22:36705329-36705441	MYH9
1198	chr22:36708096-36708267	MYH9
1199	chr22:36710192-36710363	MYH9
1200	chr22:36712564-36712714	MYH9
1201	chr22:36714254-36714370	MYH9
1202	chr22:36715585-36715682	MYH9
1203	chr22:36716267-36716408	MYH9
1204	chr22:36716845-36716941	MYH9
1205	chr22:36717787-36717884	MYH9
1206	chr22:36718472-36718570	MYH9
1207	chr22:36722612-36722709	MYH9
1208	chr22:36723472-36723569	MYH9
1209	chr22:36737417-36737571	MYH9
1210	chr22:36744951-36745281	MYH9
1211	chr22:38097375-38097486	TRIOBP

1212	chr22:38106436-38106573	TRIOBP
1213	chr22:38109219-38109418	TRIOBP
1214	chr22:38111772-38111941	TRIOBP
1215	chr22:38119194-38122510	TRIOBP
1216	chr22:38129307-38129419	TRIOBP
1217	chr22:38130408-38131449	TRIOBP
1218	chr22:38134640-38134737	TRIOBP
1219	chr22:38136904-38137039	TRIOBP
1220	chr22:38142247-38142427	TRIOBP
1221	chr22:38147760-38147857	TRIOBP
1222	chr22:38150886-38150991	TRIOBP
1223	chr22:38151105-38151202	TRIOBP
1224	chr22:38151559-38151666	TRIOBP
1225	chr22:38153622-38154145	TRIOBP
1226	chr22:38155163-38155271	TRIOBP
1227	chr22:38155426-38155534	TRIOBP
1228	chr22:38161679-38161824	TRIOBP
1229	chr22:38164083-38164183	TRIOBP
1230	chr22:38165037-38165194	TRIOBP
1231	chr22:38165271-38165382	TRIOBP
1232	chr22:38167652-38167750	TRIOBP
1233	chr22:38168610-38168769	TRIOBP
1234	chrX:9621629-9621729	TBL1X
1235	chrX:9622257-9622362	TBL1X
1236	chrX:9652085-9652228	TBL1X
1237	chrX:9656059-9656315	TBL1X
1238	chrX:9659621-9659751	TBL1X
1239	chrX:9660155-9660294	TBL1X
1240	chrX:9661173-9661270	TBL1X
1241	chrX:9661363-9661460	TBL1X
1242	chrX:9665392-9665489	TBL1X
1243	chrX:9673035-9673154	TBL1X
1244	chrX:9677278-9677375	TBL1X
1245	chrX:9677675-9677800	TBL1X
1246	chrX:9679653-9679816	TBL1X
1247	chrX:9682944-9683043	TBL1X
1248	chrX:9684226-9684323	TBL1X
1249	chrX:70443560-70444409	GJB1
1250	chrX:82763335-82764418	POU3F4
1251	chrX:100601489-100601648	TIMM8A
1252	chrX:100603335-100603432	TIMM8A
1253	chrX:100603523-100603652	TIMM8A
