# Supplementary material for: Combined examination of sequence and copy number variations in human deafness genes improves diagnosis for cases of genetic deafness
Source: BMC Ear Nose Throat Disord. 2014 Sep 10;14:9. doi: 10.1186/1472-6815-14-9 (PMC4194081; doi:10.1186/1472-6815-14-9)
Supplement: Additional file 2: Table S2 — Definition of nine categories of mutations/variants in targeted deafness genes detected by the NGS method. [file 1472-6815-14-9-S2.pdf]

Supplemental Table 2. Definition of nine categories of mutations/variants in targeted deafness genes detected by the NGS method in this study**.**

Category	Definition	Implication
I	Disease-causing mutations as suggested by reports in scientific research literature. The disease-causing nature of such mutations are also evaluated by other factors, such as allele frequencies in the general populations, inheritance patterns in known deaf families, manual evaluation of methodologies, quality of research in the published reports, whether contradictory reports are published in the literature, etc. Reported mutations with high population allele frequencies (>0.02) reported by 1000 genome project are filtered out.	disease-causing
II	Recessive disease-causing mutations as defined in the category I, exist in heterozygous format. The implication is that the patient carries recessive disease-causing mutations. Such mutations in heterozygous format may not be disease-causing, but may significantly increase the genetic risk for offspring if both parents carry the same mutations or similar mutations in the same gene. Variants with high population allele frequency (>0.1) reported by data from 1000 genome project are filtered out.	carrier of disease-causing mutations
III	Homozygous mutations not previously reported in the literature and are highly-likely to be disease-causing. These include homozygous mutations in the coding regions that cause frameshift, or stop gain/loss, or change of splicing site. Variants with high population allele frequencies (>0.02) reported by data from the 1000 genome project are filtered.	highly-likely to be disease-causing
IV	Heterozygous mutations as defined in the category III. Patient may be a carrier of such highly-likely disease-causing mutations. Such mutations in heterozygous format may not be disease-causing, but may significantly increase the genetic risk for offspring if both parents carry the same mutations or similar mutations in the same gene. Variants with high population allele frequencies (>0.1) reported by 1000 genome project are filtered.	carrier of mutations highly-likely to be disease-causing
V	Homozygous genetic mutations of unknown significance, but potentially may cause deafness. These variants are predicted computationally by two independent bioinformatic algorithms (SIFT and Polyphen2) as damaging or probably damaging on protein functions of deafness genes (which are known to be essential for hearing). Variants with high population allele frequencies (>0.02) reported by 1000 genome project are filtered out.	predicted but unconfirmed to be disease-causing
VI	Heterozygous variants as defined in the category V. Patient may be a carrier of such potential disease-causing mutations. Such mutations in heterozygous format may not be disease-causing. Variants with high population allele frequencies (>0.1) reported by 1000 genome project are filtered out.	carrier of mutations in category V
VII	Homozygous rare non-synonymous variants, in which "rare" refers to one of the following: (1) no dbSNP id found; (2) either not reported in data from the 1000 genome project, or (3) the population frequency in the 1000 genome project database is less than 0.005.	mutations of unknown significance
VIII	Heterozygous variants of those as defined in the category VII.	carrier of mutations in category VII
IX (high PAF)	Variants otherwise fit the criteria as defined for categories I-VI, but with high population allele frequency (PAF) according to data collected by the 1000 genome project (>0.02 for homozygous/dominant mutations, or >0.1 for heterozygous mutations).	
