# Supplementary material for: Combined examination of sequence and copy number variations in human deafness genes improves diagnosis for cases of genetic deafness
Source: BMC Ear Nose Throat Disord. 2014 Sep 10;14:9. doi: 10.1186/1472-6815-14-9 (PMC4194081; doi:10.1186/1472-6815-14-9)
Supplement: Additional file 3: Table S4 — Primers used in real-time qPCR validation detection of CNVs. [file 1472-6815-14-9-S3.pdf]

Sample	Gene	Position of CNVs on the gene	Length of product	Primers	
Ot3257	COL11A2	8th exon	250bps	LEFT<br>RIGHT	ccttcccactctacccatca<br>taaaggccaggaggtcagaa
Ot3255	MYO15A	44th exon	360bps	LEFT<br>RIGHT	cctttgtgaagagggctgag<br>gctcagtcttcctggcactc
Ot3255	DIAPH1	14th exon	300bps	LEFT<br>RIGHT	ctggctaaagagcgactgg<br>tgggagggacactcacaaat
Ot3272	DIAPH1	22th exon	280bps	LEFT<br>RIGHT	catctgctagatggggctgt<br>agaaagaacaggcgtccaga
Ot3270	STRC	6th exon	300bps	LEFT<br>RIGHT	ccaccatgacctcctattcg<br>ccactaaagcaagccctctg
Ot3270	STRC	17-18 exon	650bps	LEFT<br>RIGHT	ccataaattacggtggatgaa<br>ttgaataaaatcctctgctttgg
Ot3252	MYO3A	22nd exon	350bps	LEFT<br>RIGHT	tgttcactgcacattgtcctt<br>ggcggaaacagacagctatg
Ot3262	DIAPH1	26th exon	500bps	LEFT<br>RIGHT	taagggcctctgtgaagtgg<br>ggcagctgaagctgtatatg
Ot3271	COLL11A2	9th exon	250bps	LEFT<br>RIGHT	cccttcctcatctgtggtgt<br>ggcccctgagacgatactag
Ot3233	OTOA	7th exon	550bps	LEFT<br>RIGHT	tcaaactgaccctggcttct<br>cttggaattgaggcaggaga
Ot3233	OTOA	21st exon	500bps	LEFT<br>RIGHT	accccacaggatgtgtttct<br>gccaaaagaagaaaggtcaca
Ot3233	OTOA	29th exon	500bps	LEFT<br>RIGHT	gcaaagttgtgtgcatcctg<br>tccccagtgactgtcagaga
Ot3226	STRC	2nd exon	1170bps	LEFT<br>RIGHT	tttgggctctgtgttcctct<br>ccctcagaactggtctcctg
Ot3226	CATSPER	5th exon	450bps	LEFT<br>RIGHT	gacaggtgccatggttcaaa<br>gctttctctttccctctccct
Ot3226	CATSPER	2nd exon	500bps	LEFT<br>RIGHT	tccacccacaccataggatt<br>aagtgggacacattcccaga

Supplemental Table 4. Primers used in real-time qPCR detection of CNVs.
