# Supplementary material for: Combined examination of sequence and copy number variations in human deafness genes improves diagnosis for cases of genetic deafness
Source: BMC Ear Nose Throat Disord. 2014 Sep 10;14:9. doi: 10.1186/1472-6815-14-9 (PMC4194081; doi:10.1186/1472-6815-14-9)
Supplement: Additional file 4: Table S5 — Clinical findings and summary of NGS results of 79 patients. [file 1472-6815-14-9-S4.docx]

| **Sample_ID** | **Gender** | **Pure tone audiogram test results** | **Type of hearing loss** | **Age of onset** | **Use of ototoxic drugs** | **Family history** | **Progres-sive hearing loss?** | **Head Trauma ?** | **Tinnitus (which ear)** | **Vertigo?** | **Candidate mutation**  **(Category II, VI ,VI or VII)** | **Causative mutation (Category I,III or V)** | **CNV identified** |
| --- | --- | --- | --- | --- | --- | --- | --- | --- | --- | --- | --- | --- | --- |
| Ot3209 | Male | R: 30;  L: 101 | Slight | 9 | No | No | Yes | No | Yes (both) | No |  | ESRRB:NM_004452:exon4:c.A16G:p.R6G |  |
| Ot3210 | Male | R: 45;  L: 46 | Moderate | 30 | No | No | No | No | Yes (both) | No |  |  |  |
| Ot3211 | Female | R: 98;  L: 101 | Profound | 7 | Yes | No | Yes | No | No | No |  |  |  |
| Ot3212 | Female | R: 65;  L: 46 | Moderate | 0 | Not sure | No | Yes | No | No | No |  | GJB2(NM_004004:exon2: c.235delC: p. L79fs) | MYO1C  Chr17: 1388949  -1389047 Loss |
| Ot3213 | Male | R>120;  L>120 | Profound | 1 | No | No | No | No | Not sure | No | MYO7A Hom NM_001127179:exon27:c.3514_3535del:p.1172_1179del  MYO7A Hom NM_000260:exon36:c.A4996T:p.S1666C | SLC26A4(NM_000441:exon8:c.919-2A>G) | MYO6 Chr6: 76545598  -76545696 Gain   - MYO7A Chr11: 76870484   -76870582 Gain   - OTOA Chr16: 21742157   -21742255 Loss |
| Ot3214 | Male | R: 50;  L: 53 | Moderate | 14 | Not sure | No | Yes | No | Yes (both) | No |  |  |  |
| Ot3215 | Male | R: 50;  L: 40 | Slight | 11 | No | No | Yes | No | No | No |  |  |  |
| Ot3216 | Male | R: 66;  L: 65 | Moderate | 5 | No | No | No | No | No | No |  |  | ERCC2 Chr19: 45873749  -45873847 Gain |
| Ot3217 | Male | R: 31;  L: 28 | Slight | 42 | Yes | No | No | No | Yes (both) | No |  | DSPP:NM_014208:exon5:c.A3274G:p.N1092D | - STRC Chr15: 43903102   -43903200 Gain |
| Ot3218 | Male | R: 22;  L: 30 | Slight | 14 | Not sure | No | Yes | No | No | No |  |  |  |
| ot3219 | Male | R: 43;  L: 43 | Moderate | 9 | Not sure | No | No | No | No | No |  |  |  |
| Ot3220 | Female | R: 60;  L: 58 | Moderate | 23 | Yes | No | Yes | No | Yes (both) | No |  |  |  |
| Ot3221 | Female | R: 60;  L: 51 | Moderate | 14 | No | No | No | No | No | No |  |  |  |
| Ot3222 | Male | R>120;  L>120 | Profound | 1 | No | No | No | No | Not sure | No |  |  |  |
| Ot3223 | Female | R: 85;  L: 76 | Severe | 3 | Yes | No | Yes | No | No | No |  |  |  |
| Ot3224 | Female | R: 36;  L: 48 | Slight | 40 | Not sure | Not sure | Yes | No | Yes (both) | Yes |  |  |  |
| Ot3225 | Male | R>120;  L>120 | Profound | 0 | No | No | Not sure | No | No | No |  |  |  |
| Ot3226 | Female | R: 60;  L: 65 | Moderate | 10 | Yes | No | Yes | No | Yes (both) | Yes |  | DSPP:NM_014208:exon5:c.3264_3265insCGATAGCAG:p.S1088delinsSRX  TMPRSS3:NM_001256317:exon4:c.T212C:p.F71S,TMPRSS3:NM_024022:exon4:c.T212C:p.F71S,TMPRSS3:NM_032405:exon4:c.T212C:p.F71S |  |
| Ot3227 | Male | R: 40;  L: 40 | Slight | 20 | No | No | No | No | Yes (both) | No |  | GJB2:NM_004004:exon2:c.G109A:p.V37I | DIAPH1 Chr5: 140961863  -140961961 Gain |
| Ot3228 | Female | R: 46;  L: 45 | Moderate | 42 | Yes | No | Yes | No | Yes (both) | Yes |  | GJB2:NM_004004:exon2:c.G109A:p.V37I | CDH23 Chr10: 73571695  -73571793 Gain  COL11A2 Chr6: 33138082  -33138180 Gain  DIAPH1 Chr5: 140961863  -140961961 Gain  MYO1C Chr17: 1382888  -1383000 Gain |
| Ot3229 | Female | R: 90;  L: 100 | Severe | 2 | Yes | No | No | No | No | No |  |  | MYH14 Chr19: 50764734 Gain  -50764893 |
| Ot3230 | Female | R>120;  L>120 | Profound | 0 | No | No | Yes | No | No | Not sure |  | SLC26A4(NM_000441:exon8:c.919-2A>G) | STRC Chr15: 43901456-43901554 Gain |
| Ot3231 | Male | R: 27;  L: 33 | Slight | 18 | No | No | Yes | No | Yes(left) | No |  |  | COL11A2 Chr6: 33137587-33137685 Gain  ERCC2 Chr19: 45873749-45873847 Gain |
| Ot3232 | Female | R: 40;  L: 41 | Slight | 20 | No | No | Not sure | No | Yes (both) | No | MYO1C Het NM_001080779:exon7:c.G892A:p.E298K |  | - MYO1C Chr6: 33136462   -33136560 Gain  DIAPH1 Chr5: 140961863-140961961 Loss |
| Ot3233 | Male | R: 56;  L: 70 | Moderate | 3 | No | No | Not sure | No | Yes (both) | Not sure |  |  | USH1C Chr11: 17565789-17565887 Gain  DIAPH1 Chr5: 140961863-140961961 Gain  USH1C Chr11: 17544333-17544474 Gain |
| Ot3234 | Male | R: 58;  L: 58 | Moderate | 15 | Not sure | No | Yes | No | No | No | COL11A2 Het chr6:33152835 A>T |  | COL11A2 Chr6: 33136462-33136560 Loss  COL11A2 Chr6: 33138082  -33138180 Gain  DFNA5 Chr7: 24742368  -24742466 Gain  ERCC2 Chr19: 45867493  -45867592 Loss  STRC Chr15: 43900063  -43900174 Gain |
| Ot3235 | Male | R: 48;  L: 63 | Moderate | 7 | No | No | No | No | No | No |  |  | DIAPH1 Chr5: 140961863  -140961961 Gain  USH1C Chr11: 17565789  -17565887 Loss |
| Ot3236 | Female | R: 80;  L: 88 | Severe | 4 | Yes | No | Yes | No | No | No |  |  |  |
| Ot3237 | Female | R: 65;  L: 65 | Moderate | 19 | Not sure | No | No | No | Yes (both) | No | MYO3A Het NM_017433:exon30:c.C3860A:p.P1287H |  | DFNB59 Chr2: 179325077  -179325174 Gain  MYO3A Chr10: 26490167  -26490265 Gain |
| Ot3238 | Female | R: 53;  L: 47 | Moderate | 27 | Not sure | No | Yes | No | Yes (both) | No |  |  | COL11A2 Chr6: 33136462  -33136560 Gain  ERCC2 Chr19: 45873749-45873847 Gain |
| Ot3239 | Male | R: 54;  L: 58 | Moderate | 33 | No | Not sure | Yes | No | Yes (both) | No | COL11A2 Het chr6:33152835 A>T |  | CDH23 Chr10: 73550048-73550171 Gain  COL11A2 Chr6: 33136462-33136560 Gain  MYO3A Chr10: 26490167-26490265 Loss  STRC Chr15: 43901456-43901554 Loss |
| Ot3240 | Female | R>120;  L>120 | Profound | 11 | No | No | No | No | No | No |  |  | DIAPH1 Chr5: 140963693-140963793 Gain |
| Ot3241 | Male | R: 53;  L: 62 | Moderate | 16 | No | No | Yes | No | No | No | MYO1C Het NM_001080779:exon14:c.A1490G:p.E497G  MYO1C Het NM_001080779:exon6:c.G776A:p.R259Q | GJB2:NM_004004:exon2:c.G109A:p.V37I | GRHL2 Chr8: 102582547-102582645 Gain  MYO1C Chr17: 1373471-1373629 Loss  MYO1C Chr17: 1373902-1374000 Gain  MYO1C Chr17: 1382888-1383000 Gain |
| Ot3242 | Male | R: 55;  L: 79 | Moderate | not sure | Not sure | No | Yes | No | No | No | COCH Het NM_001135058:exon10:c.A1303G:p.R435G  COCH Hom NM_001135058:exon11:c.A1529G:p.K510R | COCH:NM_001135058:exon11:c.A1529G:p.K510R,COCH:NM_004086:exon12:c.A1529G:p.K510R  GJB2:NM_004004:exon2:c.G109A:p.V37I | COCH Chr14: 31354602-31354827 Gain |
| Ot3243 | Male | R>120;  L>120 | Profound | 4 | No | No | No | No | No | No |  | DSPP:NM_014208:exon5:c.3456_3457insTAGCAGCGATAGCAGCGA:p.D1152delinsDX |  |
| Ot3244 | Female | R: 60;  L: 80 | Moderate | 0 | No | Yes | Not sure | No | No | No | COL11A2 Het chr6:33152835 A>T |  | COL11A2 Chr6: 33132631-33132742 Gain  MYO1C Chr17: 1382888-1383000 Gain  OTOA Chr16: 21742157-21742255 Loss |
| Ot3246 | Male | R: 53;  L: 30 | Slight | 40 | Yes | No | Yes | No | Yes (both) | Yes | MYO7A Het NM_000260:exon20:c.G2308A:p.A770T  MYO7A Het NM_001127179:exon27:c.3514_3535del:p.1172_1179del  MYO7A Het NM_000260:exon36:c.A4996T:p.S1666C |  | MYO7A Chr11: 76870484-76870582 Gain |
| Ot3247 | Female | R: 65;  L: 65 | Moderate | 12 | No | No | Yes | No | Yes (both) | No | MYO15A Het NM_016239:exon2:c.G1783A:p.A595T  MYO15A Het NM_016239:exon2:c.T2152G:p.W718G |  | COL9A3 Chr20: 61460959-61461057 Gain  GRHL2 Chr8: 102582547-102582645 Loss  MYO15A Chr11: 17544333-17544474 Gain  USH1C Chr17: 1381912-1382030 Gain |
| Ot3248 | Male | R: 88;  L: 95 | Severe | not sure | No | No | Yes | No | Yes (both) | No |  |  |  |
| Ot3249 | Male | R: 48;  L: 63 | Moderate | 4 | No | No | No | No | No | No |  |  |  |
| Ot3250 | Male | R: 51;  L: 60 | Moderate | 6 | No | No | No | No | No | No |  |  |  |
| Ot3251 | Female | R: 51;  L: 51 | Moderate | 14 | Yes | No | Yes | No | Yes (both) | No |  |  | MYO6 Chr6: 76538246-76538344 Gain |
| Ot3252 | Male | R: 70;  L: 38 | Slight | 0 | No | No | Yes | No | Yes (both) | No |  | GJB2:NM_004004:exon2:c.G109A:p.V37I | MYO3A Chr10: 26442771-26442869 Gain |
| Ot3253 | Female | R: 51;  L: 53 | Moderate | 14 | Not sure | Not sure | Not sure | No | Yes (both) | Yes |  |  |  |
| Ot3254 | Male | R: 40;  L: 43 | Slight | 7 | No | No | Yes | No | No | No |  |  |  |
| Ot3255 | Male | R>120;  L>120 | Profound | 10 | Not sure | No | No | No | No | Not sure | MYO15A Het NM_016239:exon2:c.G1783A:p.A595T  MYO15A Het NM_016239:exon2:c.T2152G:p.W718G | GJB2:NM_004004:exon2:c.235delC:p.L79fs | DIAPH1 Chr5: 140961863-140961961 Gain  EYA4 Chr6: 133595888-133595986 Loss  MYO15A Chr17: 18067056-18067155 Gain |
| Ot3256 | Male | R: 75;  L>12  0 | Seve  re | 1 | Yes | No | Not sure | No | Not sure | Not sure | SLC26A4 Hom  NM_000441:exon8:c.919-2A>G | DSPP:NM_014208:exon5:c.3264_3265insCGATAGCAG:p.S1088delinsSRX  SLC26A4(NM_000441:exon8:c.919-2A>G) | MYO1C Chr17: 1382713-1382811 Gain  SLC26A4 Chr7: 107302089-107302251 Gain  SLC4A11 Chr20: 3210822-3210920 Gain  TMPRSS5 Chr11: 113560955-113561096 Gain |
| Ot3257 | Male | R: 75;  L: 68 | Moderate | 18 | Yes | No | No | No | Yes (both) | Yes |  |  | COL11A2 Chr6: 33139027-33139125 Loss  MYO3A Chr10: 26442771-26442869 Loss |
| Ot3258 | Female | R: 41;  L: 41 | Moderate | 2 | No | No | No | No | Yes (both) | Yes |  | SLC26A4(NM_000441:exon8:c.919-2A>G) |  |
| Ot3259 | Female | R: 56;  L: 41 | Moderate | 19 | No | No | No | No | Yes (both) | Yes | COL11A2 Het  chr6:33152835 A>T |  | COL11A2 Chr6: 33138082-33138180 Loss |
| Ot3260 | Female | R: 61;  L: 70 | Moderate | 0 | Not sure | No | No | No | No | No |  | DSPP:NM_014208:exon5:c.A3274G:p.N1092D | DIAPH1 Chr5: 140998367-140998482 Loss  MYO1C Chr17: 1388949-1389047 Loss |
| Ot3261 | Male | R: 95;  L: 95 | Profound | 12 | No | No | Yes | No | Yes (right) | No | COL11A2 Het chr6:33152835 A>T |  | COL11A2 Chr6: 33138082-33138180 Gain  MYO1C Chr17: 1388949-1389047 Gain |
| Ot3262 | Male | R: 70;  L: 70 | Moderate | 32 | Yes | No | No | No | Yes (both) | Yes |  |  | DIAPH1 Chr5: 140998367-140998482 Gain  MYO1C Chr17: 1382713-1382811 Gain |
| Ot3263 | Male | R: 95;  L: 110 | Profound | 32 | Yes | No | No | No | Yes (both) | Yes |  |  | DIAPH1 Chr5: 140998367-140998482 Gain  GRHL2 Chr8: 102676667-102676765 Gain  MYH14 Chr19: 50764734-50764893 Gain  OTOA Chr16: 21742157-21742255 Gain |
| Ot3264 | Female | R:70;  L:75 | Moderate | 7 | Not sure | No | Yes | No | No | Yes |  |  | MYO3A Chr10: 26490167-26490265 Loss  STRC Chr15: 43901456-43901554 Loss |
| Ot3265 | Female | R: 80;  L: 76 | Severe | 2 | Yes | No | No | No | No | Yes | DIAPH1 Het NM_001079812:exon15:c.T2072A:p.I691N |  | DIAPH1 Chr5: 140998367-140998482 Gain  MTAP Chr9: 21816695-21816793 Loss  MYO6 Chr6: 76545598-76545696 Gain |
| Ot3266 | Female | R>120;  L:98 | Profound | 4 | Not sure | No | Yes | No | Yes (both) | No |  | DSPP:NM_014208:exon5:c.3264_3265insCGATAGCGG:p.S1088delinsSRX  SLC26A4(NM_000441:exon8:c.919-2A>G) |  |
| Ot3267 | Female | R: 110;  L: 110 | Profound | 3 | No | No | Yes | No | No | No |  |  | DIAPH1 Chr5: 140958084-140958193 Gain  DIAPH1 Chr5: 140998367-140998482 Gain  OTOA Chr16: 21742157-21742255 Loss |
| ot3268 | Male | R: 78;  L: 75 | Severe | 0 | Not sure | No | No | No | Yes (both) | No | COL11A2  Het chr6:33152835 A>T | MYO6:NM_004999:exon17:c.A1681G:p.R561G | COL11A2 Chr6: 33138082-33138180 Gain  MYO1C Chr17: 1373902-1374000 Gain  OTOA Chr16: 21742157-21742255 Gain |
| Ot3269 | Female | R: 110;  L:110 | Profound | 7 | Not sure | No | No | No | Yes (both) | Yes |  |  |  |
| Ot3270 | Female | R: 60;  L: 58 | Moderate | 5 | Not sure | No | No | No | No | Yes |  | DSPP:NM_014208:exon5:c.3264_3265insCGATAGCAA:p.S1088delinsSRX | DIAPH1 Chr5: 140960313-140960451 Loss  STRC Chr15: 43901456-43901554 Gain |
|  |  |  |  |  |  |  |  |  |  |  |  | DSPP:NM_014208:exon5:c.A3274G:p.N1092D |  |
| Ot3271 | Male | R: 75;  L: 71 | Severe | 3 | No | No | No | No | No | No | COL11A2 Het chr6:33152835 A>T | GJB2:NM_004004:exon2:c.G109A:p.V37I | COL11A2 Chr6: 33138082-33138180 Gain |
| Ot3272 | Male | R: 48;  L: 45 | Moderate | 7 | No | No | No | No | Yes (both) | No |  |  | DIAPH1 Chr5: 140961863-140961961 Loss  MYO1C Chr17: 1388949-1389047 Gain  STRC Chr15: 43901456-43901554 Loss |
| Ot3273 | Female | R: 95;  L: 95 | Profound | 16 | Not sure | No | No | No | Yes (both) | No |  |  |  |
| Ot3274 | Female | R: 28;  L: 21 | Slight | 36 | No | No | Yes | No | Yes (both) | Yes |  |  |  |
| Ot3275 | Male | R: 78;  L: 78 | Severe | 1 | No | No | Yes | No | No | No |  | WFS1:NM_001145853:exon8:c.A2158G:p.I720V,WFS1:NM_006005:exon8:c.A2158G:p.I720V |  |
| Ot3276 | Female | R: 55;  L: 53 | Moderate | 3 | Not sure | No | No | No | No | No |  |  |  |
| Ot3277 | Male | R>120;  L>120 | Profound | 7 | No | No | Yes | No | No | No |  |  | LHX3 Chr9: 139090756-139090906 Gain |
| Ot3278 | Female | R: 80;  L: 30 | Slight | 0 | Not sure | No | No | No | No | No |  |  | OTOR Chr20: 16731704-16731802 Loss  STRC Chr15: 43901456-43901554 Gain |
| Ot3279 | Female | R: 110;  L: 113 | Profound | 1 | Not sure | No | Yes | No | Yes (both) | Yes |  |  | OTOA Chr16: 21742157-21742255 Loss |
| Ot3280 | Female | R>120;  L>120 | Profound | not sure | No | No | No | No | No | No |  |  |  |
| Ot3281 | Female | R: 60;  L: 58 | Moderate | 6 | No | No | No | No | Yes (left) | No |  |  |  |
| ot3282 | Female | R>120;  L>120 | Profound | 46 | No | Yes | Yes | No | Yes (left) | No | SOX2 Het NM_003106:exon1:c.A49G:p.T17A  SOX2 Het NM_003106:exon1:c.T73G:p.S25A  SOX2 Het NM_003106:exon1:c.G88A:p.A30T  SOX2 Het NM_003106:exon1:c.C528G:p.D176E  SOX2 Het NM_003106:exon1:c.T883G:p.S295A |  | SOX2 chr3:181430149-181431100 gain |
| Ot3283 | Female | R: 40;  L: 36 | Slight | 36 | Yes | No | Yes | No | Yes (both) | No |  |  |  |
| Ot3284 | Female | R>120;  L>120 | Profound | 2 | No | No | Yes | No | Not sure | No |  | DSPP:NM_014208:exon5:c.A3274G:p.N1092D  SLC26A4(NM_000441:exon8:c.919-2A>G)  SOX2:NM_003106:exon1:c.A49G:p.T17A |  |
| Ot3285 | Female | R:55;  L:70 | Moderate | 8 | No | No | Yes | No | Yes (left) | No | SOX2 Het NM_003106:exon1:c.A49G:p.T17A  SOX2 Het NM_003106:exon1:c.C528G:p.D176E  SOX2 Het NM_003106:exon1:c.T883G:p.S295A  GJB3 Het NM_001005752:exon2:c.G580A:p.A194T |  | SOX2 Chr3: 181430151-181431103 Gain  GJB3 chr1:35250366-35251177 loss |
| Ot3286 | Male | R:50;  L:56 | Moderate | 0 | No | Yes | No | No | Yes (left) | Yes |  |  |  |
| Ot3287 | Female | R:110;  L:90 | Severe | 1 | Not sure | No | No | No | Yes (left) | No |  |  |  |
| Ot3288 | Male | R:80;  L:80 | Severe | 0 | No | No | Yes | No | No | No |  |  |  |

Supplemental Table 5. Clinical information for 79 patients, deafness mutation(s) and CNVs identified.
