# Supplementary material for: Combined examination of sequence and copy number variations in human deafness genes improves diagnosis for cases of genetic deafness
Source: BMC Ear Nose Throat Disord. 2014 Sep 10;14:9. doi: 10.1186/1472-6815-14-9 (PMC4194081; doi:10.1186/1472-6815-14-9)
Supplement: Additional file 5: Table S3 — Comparison of CNV results obtained by NGS and qPCR methods. [file 1472-6815-14-9-S5.pdf]

Sample	Gene Name	Position of CNVs on the gene	Length of product	Value of CNV ratio obtained by NGS approach	ΔΔCq	$2^{-\Delta\Delta Cq}$	Gain/loss	Validation results
Ot3257	COL11A2	8th exon	250bps	0.53	1.56	0.34	Loss	Agree
Ot3255	MYO15A	44th exon	360bps	1.83	-0.65	1.56	Gain	Agree
Ot3255	DIAPH1	14th exon	300bps	2.76	-1	2	Gain	Agree
Ot3272	DIAPH1	22th exon	280bps	0.47	1.14	0.45	Loss	Agree
Ot3270	STRC	6th exon	300bps	1.84	-1.09	2.13	Gain	Agree
Ot3270	STRC	17-18 exon	650bps	1.82	2.12	0.23	Gain	Disagree
Ot3252	MYO3A	22nd exon	350bps	1.94	-0.74	1.89	Gain	Agree
Ot3262	DIAPH1	26th exon	500bps	2.37	1.45	2.73	Gain	Agree
Ot3271	COLL11A2	9th exon	250bps	2.09	-0.45	1.45	Gain	Agree
Ot3233	OTOA	7th exon	550bps	0.47	-0.48	0.72	Loss	Agree
Ot3233	OTOA	21st exon	500bps	0.36	-0.46	1.38	Loss	Agree
Ot3233	OTOA	29th exon	500bps	0.39	-1.33	0.4	Loss	Agree
Ot3226	STRC	2nd exon	1170bps	1.91	1.68	0.8	Gain	Agree
Ot3226	CATAPER	7nd exon	450bps	2.09	-0.31	1.24	Gain	Agree
Ot3226	CATAPER	2nd exon	500bps	2.13	-1.02	2.03	Gain	Agree

Supplemental Table 3. Comparison of CNV results obtained by NGS and qPCR methods.
