# Supplementary material for: Combined examination of sequence and copy number variations in human deafness genes improves diagnosis for cases of genetic deafness
Source: BMC Ear Nose Throat Disord. 2014 Sep 10;14:9. doi: 10.1186/1472-6815-14-9 (PMC4194081; doi:10.1186/1472-6815-14-9)
Supplement: Additional file 6: Table S6 — Information of 12 patients who showed both point mutations & CNVs. [file 1472-6815-14-9-S6.docx]

| **Sample** | **Gender** | **Pure tone audiogram test results** | **Type of hearing loss** | **Age of onset** | **Use of ototoxic drugs** | **Family history** | **Progressive hearing loss?** | **Head trauma?** | **Tinnitus (which ear)** | **Vertigo?** | **Candidate mutations** | **CNV identified** |
| --- | --- | --- | --- | --- | --- | --- | --- | --- | --- | --- | --- | --- |
| Ot3232 | Female | R: 40;  L: 41 | Slight | 20 | No | No | Not sure | No | Yes (both) | No | MYO1C Het NM_001080779:exon7:c.G892A:p.E298K | - MYO1C Chr6: 33136462   -33136560 Gain  DIAPH1 Chr5: 140961863-140961961 Loss |
| Ot3234 | Male | R: 58;  L: 58 | Moderate | 15 | Not sure | No | Yes | No | No | No | COL11A2 Het chr6:33152835 A>T | COL11A2 Chr6: 33136462-33136560 Loss  COL11A2 Chr6: 33138082  -33138180 Gain  DFNA5 Chr7: 24742368  -24742466 Gain  ERCC2 Chr19: 45867493  -45867592 Loss  STRC Chr15: 43900063  -43900174 Gain |
| Ot3237 | Female | R: 65;  L: 65 | Moderate | 19 | Not sure | No | No | No | Yes (both) | No | MYO3A Het NM_017433:exon30:c.C3860A:p.P1287H | DFNB59 Chr2: 179325077  -179325174 Gain  MYO3A Chr10: 26490167  -26490265 Gain |
| Ot3239 | Male | R: 54;  L: 58 | Moderate | 33 | No | Not sure | Yes | No | Yes (both) | No | COL11A2 Het chr6:33152835 A>T | CDH23 Chr10: 73550048-73550171 Gain  COL11A2 Chr6: 33136462-33136560 Gain  MYO3A Chr10: 26490167-26490265 Loss  STRC Chr15: 43901456-43901554 Loss |
| Ot3244 | Female | R: 60;  L: 80 | Moderate | 0 | No | Yes | Not sure | No | No | No | COL11A2 Het chr6:33152835 A>T | COL11A2 Chr6: 33132631-33132742 Gain  MYO1C Chr17: 1382888-1383000 Gain  OTOA Chr16: 21742157-21742255 Loss |
| Ot3246 | Male | R: 53;  L: 30 | Slight | 40 | Yes | No | Yes | No | Yes (both) | Yes | MYO7A Het NM_000260:exon20:c.G2308A:p.A770T  MYO7A Het NM_001127179:exon27:c.3514_3535del:p.1172_1179del  MYO7A Het NM_000260:exon36:c.A4996T:p.S1666C | MYO7A Chr11: 76870484-76870582 Gain |
| Ot3247 | Female | R: 65;  L: 65 | Moderate | 12 | No | No | Yes | No | Yes (both) | No | MYO15A Het NM_016239:exon2:c.G1783A:p.A595T  MYO15A Het NM_016239:exon2:c.T2152G:p.W718G | COL9A3 Chr20: 61460959-61461057 Gain  GRHL2 Chr8: 102582547-102582645 Loss  MYO15A Chr11: 17544333-17544474 Gain  USH1C Chr17: 1381912-1382030 Gain |
| Ot3259 | Female | R: 56;  L: 41 | Moderate | 19 | No | No | No | No | Yes (both) | Yes | COL11A2 Het  chr6:33152835 A>T | COL11A2 Chr6: 33138082-33138180 Loss |
| Ot3261 | Male | R: 95;  L: 95 | Profound | 12 | No | No | Yes | No | Yes (right) | No | COL11A2 Het chr6:33152835 A>T | COL11A2 Chr6: 33138082-33138180 Gain  MYO1C Chr17: 1388949-1389047 Gain |
| Ot3265 | Female | R: 80;  L: 76 | Severe | 2 | Yes | No | No | No | No | Yes | DIAPH1 Het NM_001079812:exon15:c.T2072A:p.I691N | DIAPH1 Chr5: 140998367-140998482 Gain  MTAP Chr9: 21816695-21816793 Loss  MYO6 Chr6: 76545598-76545696 Gain |
| Ot3282 | Female | R>120;  L>120 | Profound | 46 | No | Yes | Yes | No | Yes (left) | No | SOX2 Het NM_003106:exon1:c.A49G:p.T17A  SOX2 Het NM_003106:exon1:c.T73G:p.S25A  SOX2 Het NM_003106:exon1:c.G88A:p.A30T  SOX2 Het NM_003106:exon1:c.C528G:p.D176E  SOX2 Het NM_003106:exon1:c.T883G:p.S295A | SOX2 chr3:181430149-181431100 gain |
| Ot3285 | Female | R:55;  L:70 | Moderate | 8 | No | No | Yes | No | Yes (left) | No | SOX2 Het NM_003106:exon1:c.A49G:p.T17A  SOX2 Het NM_003106:exon1:c.C528G:p.D176E  SOX2 Het NM_003106:exon1:c.T883G:p.S295A  GJB3 Het NM_001005752:exon2:c.G580A:p.A194T | SOX2 Chr3: 181430151-181431103 Gain  GJB3 chr1:35250366-35251177 loss |

Supplemental Table 6. Information for 12 patients who are carriers of deafness mutation and also bear CNVs
